# α(δ')-Michael Addition of Alkyl Amines to Dimethyl (*E*)-hex-2-en-4-ynedioate: Synthesis of α,β-Dehydroamino Acid Derivatives

**DOI:** 10.3390/molecules18032611

**Published:** 2013-02-27

**Authors:** Arjun S. Chavan, Jie-Cheng Deng, Shih-Ching Chuang

**Affiliations:** Department of Applied Chemistry, National Chiao Tung University, Hsinchu 30010, Taiwan

**Keywords:** α-Michael addition, conjugated enyne, dehydroamino acids

## Abstract

The direct nucleophilic addition of alkyl amines to the α(δ')-carbon atom of dimethyl (*E*)-hex-2-en-4-ynedioate to generate α,β-dehydroamino acid derivatives is reported. Herein, we have studied the reactivity of various primary and secondary alkyl amines in the α-selective nucleophilic conjugate addition to conjugated dimethyl (*E*)-hex-2-en-4-ynedioate. The reaction with primary alkyl amines gives only the (2*E*,4*E*)-stereoisomer, while that with secondary alkyl amines gives the (2*E*,4*E*) and (2*Z*,4*E*)-stereoisomers of dimethyl (2-alkylamino)-muconic ester.

## 1. Introduction

The Michael addition reaction, the regioselective 1,4-addition of nucleophiles to activated olefins, is one of the versatile methods used for C–C, C–N bond formation, and has found wide application in organic synthesis [1,2,3,4,5,6,7,8,9,10]. On the other hand, the *anti*-Michael or α-Michael addition reaction, the nucleophilic addition to the α-carbon of an activated olefinic compound, is also known in the literature [11,12,13]. The regioselectivity of the reactions can be reversed by the substitution of highly electron withdrawing groups at the β-position of the Michael acceptors [14,15]. The use of phosphine bases [16,17,18], palladium catalysts [19] and organometallic reagents [20,21] are also well-known for the reversal of regioselectivity from β-carbon to α-carbon of the Michael acceptors. The theoretical and mechanical aspects of change in regioselectivity of Michael addition reaction were extensively studied by various groups [22,23,24]. Earlier, Trost *et al.* observed the phosphine-mediated α-addition of nitrogen nucleophiles to conjugated alkynoates and used it to prepare various α,β-dehydroamino acid derivatives [25]. It occurs through the zwitterionic carbenoid species formation by the attack of phosphine on the β-carbon of the allenoate substrates. In the case of the activated enyne substrate **2**, our group has reported the α-addition of phosphine and subsequent reactions of the resulting 1,3-dipolar species with various electrophilic substrates [26,27]. Although we observed the exclusive α-addition of phosphine to enyne **2** through product analysis, we still speculated about the possibility of nucleophilic addition to β-carbon of enyne **2** with other nucleophiles. In continuation of our interest in studying the reactivity of enyne **2**, herein we have shown the direct nucleophilic addition of alkyl amines to the α(δ')-carbon of enyne **2** to generate the corresponding α,β-dehydroamino acid derivatives, which are important building blocks in protein and peptide chemistry [28,29,30].

**Scheme 1:**
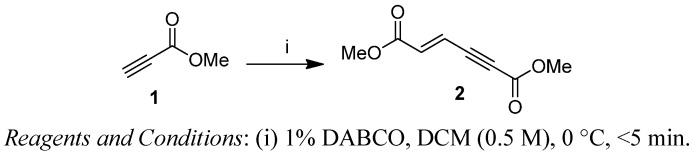
Preparation of propiolate dimer **2**.

First, we have prepared the required propiolate dimer **2** in quantitative yield by base-catalyzed dimerization of methyl propiolate (**1**) under mild reaction conditions, according to a literature procedure (Scheme 1) [31]. Recently, Pu and co-workers have shown the regio- and stereoselective addition of dibenzyl amine to propiolate dimer **2** to give exclusively the (2*E*,4*E*)-stereoisomer in quantitative yields [32]. After our success with a three-component reaction of dimer **2** with phosphines and aldehydes, we next expanded our investigation of the reaction scope of alkylamines with **2** under various reaction conditions for the regio- and chemoselective addition. Our results have shown that the produced stereoisomers are highly dependent on the degrees of substitution of the alkyl amines—primary alkyl amine gives only the (2*E*,4*E*)-stereoisomer, while secondary alkyl amines give the (2*E*,4*E*) and (2*Z*,4*E*)-stereoisomers of dimethyl (2-alkylamino)-muconic ester. All these investigated alkyl amines react with enyne **2** through α(δ')-selective nucleophilic conjugate addition.

## 2. Results and Discussion

For the reaction condition optimization study, we selected the primary amine *n*-octylamine and the secondary amine piperidine as nucleophilic partners. When we treated *n*-octylamine with propiolate dimer **2** in 1:1 molar ratio under different solvent conditions (Table 1, entry 1–6), the reaction in THF at room temperature gave stereoisomer (2*E*,4*E*)-**4** in higher yield (82%, Table 1, entry 1). Here we expected the β-addition of the nucleophile to conjugated enyne **2**, in a normal Michael addition pattern in the absence of phosphine ligand. However, this enyne diester **2** undergoes a direct nucleophilic addition to the α(δ')-carbon with no use of phosphine ligand. The unusual reactivity of this enyne diester **2** is due to the difference in electropositivity of its α(δ') and β(γ')-carbons [26].

This reaction is highly regio-, chemo- and stereoselective with primary alkyl amines. In the case of secondary amines, however, this reaction is regio- and chemoselective, but it does not show stereoselectivity since it gives (2*E*,4*E*) and (2*Z*,4*E*) stereoisomers **4** in a molar ratio range of 4:1 to 7:1 with different solvents (Table 1, entry 7–12). Our results with secondary alkyl amines are in great contrast to those of Pu’s study with dibenzylamine since we have observed two stereoisomers. Initially, we suspected the existence of β-addition product. With careful analyses by NOE experiments, we concluded that the isolated two stereoisomers are the result of α(δ')-addition and have (2*E*,4*E*) and (2*Z*,4*E*) configurations. The results obtained are summarized in Table 1. We found that the reaction carried out in THF at room temperature condition gave excellent yield for the α(δ')-addition of nitrogen nucleophiles to enyne **2**, compared to other solvent conditions.

**Table 1:** Optimization of reaction conditions ^a^.

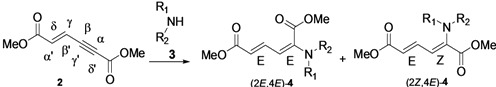					
Entry	Amine	Solvent	Reaction conditions	Yields of 4 (%) ^b^	
				(2*E*,4*E*)	(2*Z*,4*E*)
1	*n*-Octylamine	THF	rt	82	00
2	*n*-Octylamine	THF	60 °C	81	00
3	*n*-Octylamine	Benzene	rt	52	00
4	*n*-Octylamine	Toluene	rt	49	00
5	*n*-Octylamine	DCM	rt	46	00
6	*n*-Octylamine	MeCN	rt	65	00
7	Piperidine	THF	rt	64	16
8	Piperidine	THF	60 °C	54	14
9	Piperidine	Benzene	rt	68	11
10	Piperidine	Toluene	rt	60 (68)	08 (09)
11	Piperidine	DCM	rt	58 (63)	12 (13)
12	Piperidine	MeCN	rt	48 (51)	08 (09)

^a^ All reactions are carried out with 100 mg (0.595 mmol) of enyne **2** and 77 mg (0.595 mmol) of *n*-octylamine or 51 mg (0.595 mmol) of piperidine in 10 mL THF at r.t. for 2 h. ^b^ Yields were determined spectroscopically by adding mesitylene as an internal standard. Yields in parentheses are based on conversion of starting material.

Later, we used the optimized reaction conditions for studying the reactivity of propiolate dimer **2** with other primary amines **3a**–**i**. All the primary amines gave the α(δ')-addition products **4a**–**i** as a (2*E*,4*E*) stereoisomer exclusively (Table 2). Aside from *tert*-butylamine (**3g**), all other primary amines yielded the α(δ')-addition products in moderate to good yields (60–82%). The formation of α(δ')-addition products were confirmed by infrared (IR), ^1^H- and ^13^C-NMR spectroscopic methods and further supported by high resolution mass spectrometric analysis (HRMS). The ^1^H-NMR spectra of the products show doublets for the β- and δ-proton due to their couplings with the adjacent γ-proton with coupling constants of around 12 and 15 Hz, respectively. The γ-proton shows a doublet of doublet splitting due to its coupling with the β- and δ-protons, and appears in a relatively downfield shift region. The large coupling constants, ~15 Hz, between the γ- and δ-protons indicate the *trans*-configuration of the C=C double bond. In the case of piperidine, ^1^H, ^13^C and nuclear overhauser enhancement (NOE) NMR experiment confirm the formation of α(δ')-addition products (2*E*,4*E*) and (2*Z*,4*E*) as a stereoisomeric mixture (compound **4m**). In the NOE experiment of the (2*E*,4*E*)-**4m** stereoisomer, we observed enhancement in δ- and β-proton signals with about 3.0% and 2.8% respectively when we irradiated the β- and δ-protons individually. Similarly, in case of the (2*Z*,4*E*)-**4m** stereoisomer, the observed enhancements in the δ- and β-proton signals are about 2.6% and 2.2%, respectively. Later, we have carried out the reactions of secondary amines **3j**–**n** under similar reaction conditions, observing identical results with the formation of (2*E*,4*E*) and (2*Z*,4*E*) stereoisomers **4j**–**n** in 3:1 to 4:1 ratio (Table 3). The purification of primary alkyl amine products were carried out on silica gel column with hexanes/ethyl acetate mixtures as an eluent, but, in case of secondary alkyl amine products, the better separation of (2*E*,4*E*) and (2*Z*,4*E*) stereoisomers were achieved using 5% (v/v) triethylamine/hexanes on a neutralized silica gel column.

**Table 2:** Reaction of enyne **2** with primary alkyl amines ^a^.

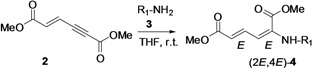			
Entry	R_1_	Product (2*E*,4*E*)	Yield (%) ^b^
1	*n*-Hexyl-**3a**	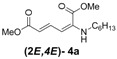	67
2	*n*-Octyl-**3b**	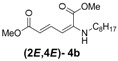	82
3	*n*-Decyl-**3c**	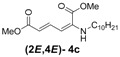	73
4	*n*-Dodecyl-**3d**	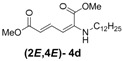	73
5 ^c^	Benzyl- **3e**	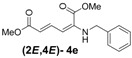	73 (75) ^c^
6	*iso*-Propyl-**3f**	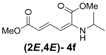	60 (64)
7	*tert*-Butyl-**3g**	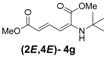	25 (58)
8	Allyl- **3h**	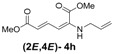	68
9	Ethanol- **3i**	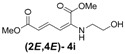	78 (80)

^a^ All reactions are carried out with 200 mg (1.19 mmol) of enyne **2** and 1.19 mmol of amine in 10 mL THF at r.t. for 2 h. ^b^ Yields were determined spectroscopically by adding mesitylene as an internal standard. Yields in parentheses are based on conversion of starting materials. ^c^ Reference [32].

**Table 3:** Reaction of enyne **2** with secondary alkyl amines ^a^.

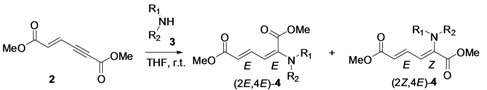						
Entry	R_1_R_2_	Major Product (2*E*,4*E*)	Yield (%) ^b^	Minor Product (2*Z*,4*E*)	Yield (%) ^b^	Ratio of (2*E*,4*E*)/(2*Z*,4*E*)
1	Diethyl **3j**	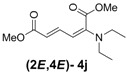	53 (58)	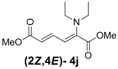	14 (15)	3.8:1
2	Dipropyl **3k**	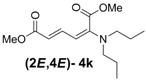	59 (62)	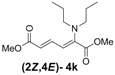	16 (17)	3.7:1
3	Pyrrolidine **3l**	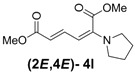	56	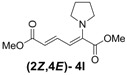	18	3.1:1
4	Piperidine **3m**	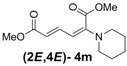	64	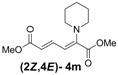	16	4:1
5	Morpholine **3n**	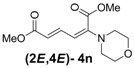	58	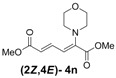	18	3.2:1

^a^ All reactions are carried out with 500 mg (2.98 mmol) of enyne **2** and 2.98 mmol of amines in 20 mL THF at r.t. for 2 h. ^b^ Yields were determined spectroscopically by adding mesitylene as an internal standard. Yields in parentheses are based on conversion of starting materials.

**Scheme 2:**
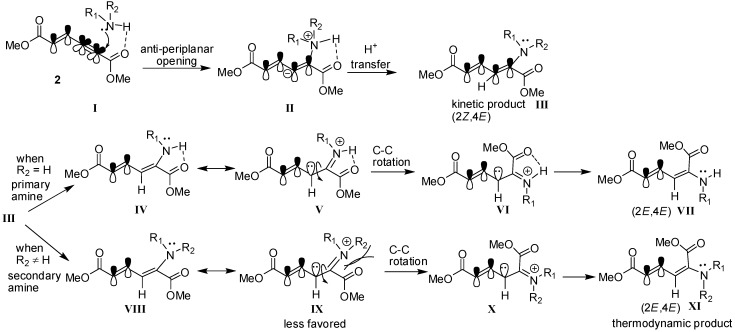
Proposed mechanism for regio- and chemoselective addition of amine to enyne **2**.

The possible mechanism for the formation of (2*E*,4*E*) and (2*Z*,4*E*)-stereoisomers is depicted in Scheme 2. Initially, amine hydrogen coordinates to the carbonyl group of alkynyl end and then nitrogen attacks the α(δ**'**)-carbon in antiperiplanar fashion to form the five membered-ring structure **II**. Later, proton transfer takes place to give the kinetic (2*Z*,4*E*)-stereoisomer product **III**. In the case of reactions with primary amines (R_2_ = H), the intramolecularly hydrogen-bonded intermediate **IV** exists as a resonance structure that can undergo C-C bond rotation to give intermediate **VI**. After resonance takes place again, the thermodynamically favored product (2*E*,4*E*)-stereoisomer forms. However, in the case of reactions with secondary amines, the steric hindrance makes the resonance structure **IX** higher in energy. Therefore, its isomerization to the thermodynamic product **XI** is relatively disfavored and slow.

## 3. Experimental

### 3.1. General

All reactions were performed under nitrogen. Anhydrous THF, benzene, and toluene were distilled over sodium/benzophenone under argon. Anhydrous DCM and MeCN were distilled over CaH_2_ under argon. The chemical shifts in the ^1^H (300 MHz) and ^13^C-NMR (75 MHz) spectra are referenced to TMS or CHCl_3_. NMR, FT-IR and mass data were recorded with Bruker DRX-300, Digilab Excalibur HE series FTS 3100 and Varian 901-MS spectrometers, respectively.

### 3.2. General Experimental Procedure for Reactions with Primary Alkyl Amines

A solution of dimer **2** (0.20 g, 1.19 mmol) in anhydrous benzene (10 mL × 4 times) was distilled from a Dean-Stark apparatus to remove the moisture residue as an azeotropic mixture. Then, 10 mL of anhydrous THF was added at room temperature followed by the addition of the appropriate amine **3a**–**i** (1.19 mmol, 1 equiv.). Then, the resulting reaction mixture was stirred for 2 h at room temperature. After completion of the reaction (TLC monitoring), the solvent was removed under vacuum and the crude product was subjected to silica gel chromatography. Elution of the column with hexanes/ethyl acetate mixture gives the yellow colored products.

*(2E,4E)-Dimethyl-2-(hexylamino)hexa-2,4-dienedioate* (**2*E***,**4*E*-4a**). *R*_f_ = 0.39 (3:1 hexanes/ethyl acetate), Yellow solid. mp: 34–36 °C; ^1^H-NMR (CDCl_3_) δ 0.88–0.92 (m, 3H), 1.26–1.43(m, 6H), 1.57–1.67 (m, 2H), 3.02 (dd, *J* = 6.9, 12.4 Hz, 2H), 3.73 (s, 3H), 3.92 (s, 3H), 4.91 (s, 1H), 5.46 (d, *J* = 12.1 Hz, 1H), 5.72 (d, *J* = 14.9 Hz, 1H), 8.23 (dd, *J* = 12.1, 14.9 Hz, 1H) ppm; ^13^C-NMR (CDCl_3_) δ 13.9, 22.5, 26.7, 28.3, 31.4, 43.3, 51.1, 52.8, 102.0, 115.3, 139.2, 142.7, 164.6, 168.4; FT-IR (KBr) (cm^–1^) 1602, 1710, 1735, 2859, 2931, 2954; HRMS (EI^+^), calcd for C_14_H_23_NO_4_ (M^+^) 269.1627 found 269.1629.

*(2E,4E)-Dimethyl-2-(octylamino)hexa-2,4-dienedioate* (**2*E***,**4*E*-4b**). *R*_f_ = 0.33 (4:1 hexanes/ethyl acetate), Yellow solid. mp: 44–46 °C; ^1^H-NMR (CDCl_3_) δ 0.86–0.91 (m, 3H), 1.28–1.40 (m, 10H), 1.57–1.64 (m, 2H), 3.01 (dd, *J* = 6.9, 12.4 Hz, 2H), 3.72 (s, 3H), 3.91 (s, 3H), 4.95 (t, *J* = 4.8 Hz, 1H), 5.46 (d, *J* = 12.1 Hz, 1H), 5.72 (d, *J* = 14.9 Hz, 1H), 8.23 (dd, *J* = 12.1, 14.9 Hz, 1H); ^13^C-NMR (CDCl_3_) δ 13.9, 22.4, 26.9, 28.1, 29.1, 29.3, 31.6, 43.1, 50.8, 52.5, 101.7, 114.9, 139.1, 142.6, 164.3, 168.2; FT-IR (KBr) (cm^–1^) 1603, 1713, 1737, 2856, 2928; HRMS (EI^+^), calcd for C_16_H_27_NO_4_ (M^+^) 297.1940 found 297.1945.

*(2E,4E)-Dimethyl-2-(decylamino)hexa-2,4-dienedioate* (**2*E***,**4*E*-4c**). *R*_f_ = 0.30 (5:1 hexanes/ethyl acetate), Yellow solid. mp: 52–54 °C; ^1^H-NMR (CDCl_3_) δ 0.86–0.90 (m, 3H), 1.27–1.36 (m, 14H), 1.57–1.66 (m, 2H), 3.01 (dd, *J* = 6.8, 12.4 Hz, 2H), 3.72 (s, 3H), 3.90 (s, 3H), 4.98 (t, *J* = 4.9 Hz, 1H), 5.45 (d, *J* = 12.1 Hz, 1H), 5.71 (d, *J* =14.9 Hz, 1H), 8.23 (dd, *J* = 12.1, 14.9 Hz, 1H); ^13^C-NMR (75.5 MHz, CDCl_3_) δ 13.9, 22.4, 26.9, 28.1, 29.1, 29.3, 31.6, 43.1, 50.8, 52.5, 101.7, 114.9, 139.1, 142.6, 164.3, 168.2; FT-IR (KBr) (cm^–1^) 1602, 1711, 1736, 2855, 2927; HRMS (EI^+^), calcd for C_18_H_31_NO_4_ (M^+^) 325.2253 found 325.2252.

*(2E,4E)-Dimethyl-2-(dodecylamino)hexa-2,4-dienedioate* (**2*E***,**4*E*-4d**). *R*_f_ = 0.35 (5:1 hexanes/ethyl acetate), Yellow solid. mp: 63–65 °C; ^1^H-NMR (CDCl_3_) δ 0.86–0.90 (m, 3H), 1.26–1.33 (m, 18H), 1.57–1.64 (m, 2H), 3.01 (dd, *J* = 6.7, 12.4 Hz, 2H), 3.72 (s, 3H), 3.91 (s, 3H), 4.96 (t, *J* = 4.7 Hz, 1H), 5.45 (d, *J* = 12.1 Hz, 1H), 5.71 (d, *J* =14.9 Hz, 1H), 8.23 (dd, *J* = 12.1, 14.9 Hz, 1H); ^13^C-NMR (CDCl_3_) δ 13.9, 22.5, 27.0, 28.2, 29.1, 29.2, 29.35, 29.41, 29.46, 29.48, 31.7, 43.2, 50.9, 52.6, 101.8, 115.0, 139.1, 142.7, 164.4, 168.3; FT-IR (KBr) (cm^–1^) 1602, 1714, 1736, 2854, 2926; HRMS (EI^+^), calcd for C_20_H_35_NO_4_ (M^+^) 353.2566 found 353.2558.

*(2E,4E)-Dimethyl-2-(isopropylamino)hexa-2,4-dienedioate* (**2*E***,**4*E*-4f**). *R*_f_ = 0.33 (4:1 hexanes/ethyl acetate), Yellow sticky liquid; ^1^H-NMR (CDCl_3_) δ 1.21 (d, *J* = 6.3 Hz, 6H), 3.46–3.57 (m, 1H), 3.72 (s, 3H), 3.91 (s, 3H), 4.86 (d, *J* = 7.1 Hz, 1H), 5.47 (d, *J* = 12.1 Hz, 1H), 5.70 (d, *J* = 14.9 Hz, 1H), 8.23 (dd, *J* = 12.1, 14.9 Hz, 1H); ^13^C-NMR (CDCl_3_) δ 21.7, 43.7, 51.0, 52.7, 101.9, 114.7, 138.1, 142.8, 164.6, 168.3; FT-IR (KBr) (cm^–1^) 1600, 1712, 2953; HRMS (EI^+^), calcd for C_11_H_17_NO_4_ (M^+^) 227.1158 found 227.1156.

*(2E,4E)-Dimethyl-2-(tert-butylamino)hexa-2,4-dienedioate* (**2*E***,**4*E*-4g**). *R*_f_ = 0.32 (4:1 hexanes/ethyl acetate), Yellow sticky liquid; ^1^H-NMR (CDCl_3_) δ 1.36 (s, 9H), 3.72 (s, 3H), 3.91 (s, 3H), 4.92 (s, 1H), 5.68 (d, *J* = 1.5 Hz, 1H), 5.73 (s, 1H), 8.14 (dd, *J* = 11.8, 15.1 Hz, 1H); ^13^C-NMR (CDCl_3_) δ 28.7, 51.0, 51.2, 52.8, 103.7, 114.7, 137.9, 143.0, 165.4, 168.4; FT-IR (KBr) (cm^–1^) 1598, 1711, 1734, 2954, 2978; HRMS (EI^+^), calcd for C_12_H_19_NO_4_ (M^+^) 241.1314 found 241.1311.

*(2E,4E)-Dimethyl-2-(allylamino)hexa-2,4-dienedioate* (**2*E***,**4*E*-4h**). *R*_f_ = 0.37 (3:1 hexanes/ethyl acetate), Yellow solid. mp: 38–40 °C; ^1^H-NMR (CDCl_3_) δ 3.67–3.69 (m, 2H), 3.72 (s, 3H), 3.92 (s, 3H), 5.14 (s, 1H), 5.20–5.29 (m, 2H), 5.49 (d, *J* = 12.0 Hz, 1H), 5.72 (d, *J* = 15.0 Hz, 1H), 5.80–5.93 (m, 1H), 8.22 (dd, *J* = 12.1, 14.9 Hz, 1H); ^13^C-NMR (CDCl_3_) δ 45.6, 51.1, 52.7, 102.9, 115.8, 117.2, 132.7, 138.6, 142.4, 164.3, 168.2; FT-IR (KBr) (cm^–1^) 1600, 1713, 1734, 2850, 2952, 3082; HRMS (EI^+^), calcd for C_11_H_15_NO_4_ (M^+^) 225.1001 found 225.0997.

*(2E,4E)-Dimethyl-2-(2-hydroxyethylamino)hexa-2,4-dienedioate* (**2*E***,**4*E*-4i**). *R*_f_ = 0.18 (1:1 hexanes/ethyl acetate), Yellow sticky liquid; ^1^H-NMR (CDCl_3_) δ 3.22 (q, *J* = 5.3 Hz, 2H), 3.73 (s, 3H), 3.86 (t, *J* = 5.2 Hz, 2H), 3.91 (s, 3H), 5.30 (s, 1H), 5.50 (d, *J* = 12.0 Hz, 1H), 5.70 (d, *J* = 15.0 Hz, 1H), 8.23 (dd, *J* = 12.0, 15.0 Hz, 1H); ^13^C-NMR (CDCl_3_) δ 45.3, 51.3, 52.8, 60.0, 102.6, 115.7, 139.3, 142.6, 164.4, 168.6; FT-IR (KBr) (cm^–1^) 1600, 1712, 2953; HRMS (EI^+^), calcd for C_10_H_15_NO_5_ (M^+^) 229.0950 found 229.0946.

### 3.3. General Experimental Procedure for Reactions with Secondary Alkyl Amines

A solution of dimer **2** (0.50 g, 2.98 mmol) in anhydrous benzene (20 mL × 3 times) was distilled from a Dean-Stark apparatus to remove the moisture residue as an azeotropic mixture. Then, anhydrous THF (10 mL) was added at room temperature, followed by the addition of the appropriate amine **3j**–**n** (2.98 mmol, 1 equiv.) Then, the resulting reaction mixture was stirred for 2 h at room temperature. After completion of the reaction (TLC monitoring), the solvent was removed under vacuum and the crude product was subjected to 5% triethylamine-neutralized silica gel chromatography to separate both the isomeric products. Elution of the column with hexanes/ethyl acetate mixture gives the corresponding yellow colored products.

*(2E,4E)-Dimethyl-2-(diethylamino)hexa-2,4-dienedioate* (**2*E***,**4*E*-4j**). *R*_f_ = 0.21 (4:1 hexanes/ethyl acetate), Yellow sticky liquid; ^1^H-NMR (CDCl_3_) δ 1.15 (t, *J* = 6.9 Hz, 6H), 3.15 (q, *J* = 6.9 Hz, 4H), 3.68 (s, 3H), 3.91 (s, 3H), 5.21 (d, *J* = 12.0 Hz, 1H), 5.56 (d, *J* = 14.4 Hz, 1H), 7.24 (dd, *J* = 12.0, 14.7 Hz, 1H); ^13^C-NMR (CDCl_3_) δ 12.9, 44.9, 51.1, 52.7, 97.2, 110.1, 143.0, 148.8, 166.0, 168.6; FT-IR (KBr) (cm^–1^) 1438, 1577, 1719, 2873, 2954, 2972; HRMS (EI^+^), calcd for C_12_H_19_NO_4_ (M^+^) 241.1314 found 241.1316.

*(2Z,4E)-Dimethyl-2-(diethylamino)hexa-2,4-dienedioate* (**2*Z***,**4*E*-4j**). *R*_f_ value = 0.47 (4:1 hexanes/ethyl acetate), Yellow sticky liquid; ^1^H-NMR (CDCl_3_) δ 1.07 (t, *J* = 7.1 Hz, 6H), 3.14 (q, *J* = 7.1 Hz, 4H), 3.74 (s, 3H), 3.78 (s, 3H), 5.91 (d, *J* = 15.3 Hz, 1H), 6.34 (d, *J* = 12.0 Hz, 1H), 7.69 (dd, *J* = 11.7, 15.3 Hz, 1H); ^13^C-NMR (CDCl_3_) δ 13.8, 47.5, 51.6, 52.2, 120.2, 120.9, 140.2, 144.7, 166.7, 167.7; FT-IR (KBr) (cm^–1^) 1436, 1578, 1725, 2872, 2954, 2972; HRMS (EI^+^), calcd for C_12_H_19_NO_4_ (M^+^) 241.1314 found 241.1315.

*(2E,4E)-Dimethyl-2-(dipropylamino)hexa-2,4-dienedioate* (**2*E***,**4*E*-4k**). *R*_f_ = 0.44 (4:1 hexanes/ethyl acetate), Yellow sticky liquid; ^1^H-NMR (CDCl_3_) δ 0.87 (t, *J* = 7.5 Hz, 6H), 1.51–1.64 (m, 4H), 3.02 (t, *J* = 7.5 Hz, 4H), 3.67 (s, 3H), 3.89 (s, 3H), 5.15 (d, *J* = 12.0 Hz, 1H), 5.40 (d, *J* = 14.7 Hz, 1H), 7.21 (dd, *J* = 12.0, 14.7 Hz, 1H); ^13^C-NMR (CDCl_3_) δ 11.4, 20.9, 51.1, 52.7, 52.9, 97.4, 111.1, 143.1, 149.3, 166.1, 168.7; FT-IR (KBr) (cm^–1^) 1458, 1522, 1725, 2857, 2926, 2956; HRMS (EI^+^), calcd for C_14_H_23_NO_4_ (M^+^) 269.1627 found 269.1628.

*(2Z,4E)-Dimethyl-2-(dipropylamino)hexa-2,4-dienedioate* (**2*Z***,**4*E*-4k**). *R*_f_ = 0.56 (4:1 hexanes/ethyl acetate), Yellow sticky liquid; ^1^H-NMR (CDCl_3_) δ 0.80 (t, *J* = 7.5 Hz, 6H), 1.37–1.49 (m, 4H), 3.00 (t, *J* = 7.3 Hz, 4H), 3.67 (s, 3H), 3.71 (s, 3H), 5.82 (d, *J* = 15.3 Hz, 1H), 6.16 (d, *J* = 12.0 Hz, 1H), 7.60 (dd, *J* = 12.0, 15.3 Hz, 1H); ^13^C-NMR (CDCl_3_) δ 11.5, 21.9, 51.6, 52.3, 55.4, 118.1, 120.3, 140.4, 145.6, 166.8, 167.7; FT-IR (KBr) (cm^–1^) 1434, 1577, 1603, 1718, 2875, 2961; HRMS (EI^+^), calcd for C_14_H_23_NO_4_ (M^+^) 269.1627 found 269.1626.

*(2E,4E)-Dimethyl-2-(pyrrolidin-1-yl)hexa-2,4-dienedioate* (**2*E***,**4*E*-4l**). *R*_f_ = 0.31 (4:1 hexanes/ethyl acetate), Yellow solid. mp: 102–104 °C; ^1^H-NMR (CDCl_3_) δ 1.90–1.95 (m, 4H), 3.21–3.25 (m, 4H), 3.67 (s, 3H), 3.90 (s, 3H), 5.14 (d, *J* = 12.0 Hz, 1H), 5.56 (d, *J* = 14.7 Hz, 1H), 7.342 (dd, *J* = 12.0, 14.7 Hz, 1H); ^13^C-NMR (CDCl_3_) δ 25.3, 48.7, 51.1, 52.8, 98.1, 111.1, 142.9, 146.7, 165.5, 168.3; FT-IR (KBr) (cm^–1^) 1438, 1569, 1697, 1733, 2867, 2952; HRMS (EI^+^), calcd for C_12_H_17_NO_4_ (M^+^) 239.1158 found 239.1167.

*(2Z,4E)-Dimethyl-2-(pyrrolidin-1-yl)hexa-2,4-dienedioate* (**2*Z***,**4*E*-4l**). *R*_f_ = 0.44 (4:1 hexanes/ethyl acetate), Yellow solid. mp: 61–63 °C; ^1^H-NMR (CDCl_3_) δ 1.88–1.92 (m, 4H), 3.51–3.55 (m, 4H), 3.69 (s, 3H), 3.79 (s, 3H), 5.56 (d, *J* = 12.9 Hz, 1H), 5.25 (d, *J* = 14.7 Hz, 1H), 7.82 (dd, *J* = 12.9, 14.4 Hz, 1H); ^13^C-NMR (CDCl_3_) δ 25.7, 51.3, 52.2, 52.6, 101.8, 114.7, 141.8, 142.3, 166.8, 168.7; FT-IR (KBr) (cm^–1^) 1452, 1575, 1704, 1728,2873, 2952; HRMS (EI^+^), calcd for C_12_H_17_NO_4_ (M^+^) 239.1158 found 239.1157.

*(2E,4E)-Dimethyl-2-(piperidin-1-yl)hexa-2,4-dienedioate* (**2*E***,**4*E*-4m**). *R*_f_ = 0.44 (3:1 hexanes/ethyl acetate), Yellow solid. mp: 68–70 °C; ^1^H-NMR (CDCl_3_) δ 1.61 (m, 6H), 3.03–3.05 (m, 4H), 3.69 (s, 3H), 3.90 (s, 3H), 5.41 (d, *J* = 11.7 Hz, 1H), 5.65 (d, *J* = 14.7 Hz, 1H), 7.68 (dd, *J* = 12.0, 14.7 Hz, 1H); ^13^C-NMR (CDCl_3_) δ 23.6, 24.9, 48.8, 50.7, 52.3, 100.8, 113.1, 142.2, 149.6, 165.7, 167.8; FT-IR (KBr) (cm^–1^) 1437, 1576, 1606, 1706, 1735, 2855, 2947; HRMS (EI^+^), calcd for C_13_H_19_NO_4_ (M^+^) 253.1314 found 253.1321.

*(2Z,4E)-Dimethyl-2-(piperidin-1-yl)hexa-2,4-dienedioate* (**2*Z***,**4*E*-4m**). *R*_f_ = 0.56 (3:1 hexanes/ethyl acetate), Yellow solid. mp: 38–40 °C; ^1^H-NMR (CDCl_3_) δ 1.62 (bs, 6H), 3.15–3.17 (m, 4H), 3.74 (s, 3H), 3.79 (s, 3H), 5.84 (d, *J* = 15.0 Hz, 1H), 6.08 (d, *J* = 12.3 Hz, 1H), 7.67 (dd, *J* = 12.0, 15.0 Hz, 1H); ^13^C-NMR (CDCl_3_) δ 24.1, 26.6, 51.6, 52.5, 53.1, 114.3, 119.6, 140.5, 146.1, 166.9, 167.9; FT-IR (KBr) (cm^–1^) 1435, 1578, 1605, 1716, 2854, 2945; HRMS (EI^+^), calcd for C_13_H_19_NO_4_ (M^+^) 253.1314 found 253.1321.

*(2E,4E)-Dimethyl-2-(morpholino)hexa-2,4-dienedioate* (**2*E***,**4*E*-4n**). *R*_f_ = 0.20 (2:1 hexanes/ethyl acetate), Yellow sticky liquid; ^1^H-NMR (CDCl_3_) δ 3.02 (t, *J* = 4.8 Hz, 4H), 3.70 (s, 3H), 3.76 (t, *J* = 4.8 Hz, 4H), 3.90 (s, 3H), 5.47 (d, *J* = 11.7 Hz, 1H), 5.73 (d, *J* = 15.0 Hz, 1H), 7.36 (dd, *J* = 11.7, 15.3 Hz, 1H); ^13^C-NMR (CDCl_3_) δ 48.4, 51.5, 52.9, 66.2, 104.6, 116.7, 141.4, 149.0, 165.6, 167.9; FT-IR (KBr) (cm^–1^) 1435, 1582, 1611, 1704, 1731, 2857, 2954; HRMS (EI^+^), calcd for C_12_H_17_NO_5_ (M^+^) 255.1107 found 255.1109.

*(2Z,4E)-Dimethyl-2-(morpholino)hexa-2,4-dienedioate* (**2*Z***,**4*E*-4n**). *R*_f_ = 0.33 (2:1 hexanes/ethyl acetate), Yellow solid. mp: 43–45 °C; ^1^H-NMR (CDCl_3_) δ 3.16 (t, *J* = 4.5 Hz, 4H), 3.74 (s, 3H), 3.75 (t, *J* = 4.5 Hz, 4H), 3.78 (s, 3H), 5.94 (d, *J* = 15.3 Hz, 1H), 6.35 (d, *J* = 12.0 Hz, 1H), 7.66 (dd, *J* = 12.0, 15.3 Hz, 1H); ^13^C-NMR (CDCl_3_) δ 51.8, 51.9, 52.5, 67.4, 119.3, 122.2, 139.3, 144.8, 166.1, 167.4; FT-IR (KBr) (cm^–1^) 1435, 1583, 1605, 1717, 2851, 2954; HRMS (EI^+^), calcd for C_12_H_17_NO_5_ (M^+^) 255.1107 found 255.1103.

## 4. Conclusions

Herein we have shown the direct and exclusive α-addition of various primary and secondary alkyl amines to a conjugated enyne. The observed addition pattern is same as that observed with phosphine nucleophiles, however, the reaction with primary alkyl amines gives the corresponding (2*E*,4*E*)-stereoisomer exclusively, and that with secondary alkyl amines gives a mixture of (2*E*,4*E*) and (2*Z*,4*E*)-stereoisomers. This direct nucleophilic addition to an activated enyne substrate is highly regio- and chemoselective. The resulting α,β-dehydroamino acid derivatives may find significant applications in protein and peptide chemistry.

## Supplementary Materials

Supplementary materials include the copies of the ^1^H-NMR and ^13^C-NMR spectra for all new compounds, which can be accessed at: http://www.mdpi.com/1420-3049/18/3/2611/s1.

## References

[1] March J. (1992). Advanced Organic Chemistry.

[2] Perlmutter P. (1992). Conjugate Addition Reactions in Organic Synthesis.

[3] Jung M.E., Trost B.M., Fleming I., Semmelhack M.F. (1991). Comprehensive Organic Synthesis.

[4] Imanzadeh G., Ahmadi F., Zamanloo M., Mansoori Y. (2010). Tetrabutylammonium Bromide Media Aza-Michael Addition of 1,2,3,6-Tetrahydrophthalimide to Symmetrical Fumaric Esters and Acrylic Esters under Solvent-Free Conditions. Molecules.

[5] Jiang Z.-Y., Yang H.-M., Ju Y.-D., Li L., Luo M.-X., Lai G.-Q., Jiang J.-X., Xu L.-W. (2010). Organocatalytic Michael Addition of 1,3-Dicarbonyl Indane Compounds to Nitrostyrenes. Molecules.

[6] Wang Y., Yuan Y.-Q., Guo S.-R. (2009). Silica Sulfuric Acid Promotes Aza-Michael Addition Reactions under Solvent-Free Condition as a Heterogeneous and Reusable Catalyst. Molecules.

[7] Escalante J., Carrillo-Morales M., Linzaga I. (2008). Michael Additions of Amines to Methyl Acrylates Promoted by Microwave Irradiation. Molecules.

[8] Chen H., Zhong X., Wei J. (2007). Stereoselective Syntheses of Fluorescent Non-Natural Aromatic Amino Acids Based on Asymmetric Michael Additions. Molecules.

[9] Davies S.G., Lee J.A., Roberts P.M., Thomson J.E., Yin J. (2011). Double Asymmetric Induction as a Mechanistic Probe: The Doubly Diastereoselective Conjugate Addition of Enantiopure Lithium Amides to Enantiopureα,β-Unsaturated Esters and Enantiopureα,β-Unsaturated Hydroxamates. Tetrahedron.

[10] Davies S.G., Smith A.D., Price P.D. (2005). The Conjugate Addition of Enantiomerically Pure Lithium Amides as Homochiral Ammonia Equivalents: Scope, Limitations and Synthetic Applications. Tetrahedron Asymmetry.

[11] Lewandowska E. (2007). Substitution at the α-Carbons of α,β-Unsaturated Carbonyl Compounds: *Anti*-Michael Addition. Tetrahedron.

[12] Bi X., Zhang J., Liu Q., Tan J., Li B. (2007). IntramolecularAza-*Anti*-Michael Addition of an Amide Anion to Enones: A Regiospecific Approach to Tetramic Acid Derivatives. Adv. Synth. Catal..

[13] Li Y., Xu X., Tan J., Liao P., Zhang J., Liu Q. (2010). Polarity-Reversible Conjugate Addition Tuned by Remote Electronic Effects. Org. Lett..

[14] Ballini R., Bazán N.A., Bosica G., Palmieri A. (2008). Uncatalyzed, *Anti*-Michael Addition of Amines to β-Nitroacrylates: Practical, Eco-Friendly Synthesis of β-Nitro-α-Amino Esters. Tetrahedron Lett..

[15] Ballini R., Bosica G., Palmieri A., Bakhtiari K. (2009). Solvent-Free, Anti-Michael Addition of Active Methylene Derivatives to β-Nitroacrylates: Eco-Friendly, Chemoselective Synthesis of PolyfunctionalizedNitroalkanes. Synlett.

[16] Wilson J.E., Sun J., Fu G.C. (2010). StereoselectivePhosphine-Catalyzed Synthesis of Highly Functionalized Diquinanes. Angew.Chem. Int. Ed. Engl..

[17] Zhu X.-F., Henry C.E., Kwon O. (2007). Stable Tetravalent PhosphoniumEnolate Zwitterions. J. Am. Chem. Soc..

[18] Lecerclé D., Sawicki M., Taran F. (2006). Phosphine-Catalyzed α-*P*-Addition on Activated Alkynes: A new route to P−C−P backbones. Org. Lett..

[19] Shim J.-G., Park J.C., Cho C.S., Shim S.C., Yamamoto Y. (2002). Catalytic and Highly Regiospecific Carbon–Carbon Bond Formation at α-Position of Michael Acceptor by Palladium Complex. Chem. Commun..

[20] Klumpp G.W., Mierop A.J.C., Vrielink J.J., Brugman A., Schakel M. (1985). *Anti*-Michael Carbolithiation of Silicon and Phenyl-Substituted α,β-Unsaturated Secondary Amides. J. Am. Chem. Soc..

[21] Aurell M.J., Bañuls M.J., Mestres R., Muñoz E. (2001). On the Mechanism of the Addition of Organolithium Reagents to Cinnamic Acids. Tetrahedron.

[22] Chatfield D.C., Augsten A., D’Cunha C., Lewandowska E., Wnuk S.F. (2004). Theoretical and Experimental Study of the Regioselectivity of Michael Additions. Eur. J. Org. Chem..

[23] Lewandowska E., Chatfield D.C. (2005). Regioselectivity of Michael Additions to 3-(Pyridin-3-yl or Pyrimidin-2-yl)-Propenoates and their *N*-Oxides—Experimental and Theoretical Studies. Eur. J. Org. Chem..

[24] Yuan H., Zheng Y., Zhang J. (2012). Mechanism Study of the Intramolecular *Anti*-Michael Addition of *N*-Alkylfurylacrylacetamides. J. Org. Chem..

[25] Trost B.M., Dake G.R. (1997). Nucleophilic α-Addition to Alkynoates. A Synthesis of Dehydroamino Acids. J. Am. Chem. Soc..

[26] Deng J.-C., Chuang S.-C. (2011). Three-Component and Nonclassical Reaction of Phosphines with Enynes and Aldehydes: Formation of γ-Lactones Featuring α-Phosphorus Ylides. Org. Lett..

[27] Chuang S.-C., Deng J.-C., Chan F.-W., Chen S.-Y., Huang W.-J., Lai L.-H., Rajeshkumar V. (2012). [3+2] Cycloaddition of Dialkyl (*E*)-Hex-2-en-4-ynedioates to [60]Fullerene by Phosphane-Promoted Tandem α(d')-Michael Additions. Eur. J. Org. Chem..

[28] Kazmaier U., Hughes A.B. (2009). Amino Acids, Peptides and Proteins in Organic Chemistry.

[29] Mathur P., Ramakumar S., Chauhan V.S. (2004). Peptide Design Using α,β-Dehydroamino Acids: From β-Turns to Helical Hairpins. Pept. Sci..

[30] Ferreira P.M.T., Maia H.L.S., Monteiro L.S., Sacramento J. (1999). High Yielding Synthesis of Dehydroamino Acid and Dehydropeptide Derivatives. J. Chem. Soc. Perkin Trans. 1.

[31] Ramachandran P.V., Rudd M.T., Reddy M.V.R. (2005). Stereoselective Synthesis of Hex-2-(*E*)-en-4-yn-1,6-dioates and *E,Z*-Muconic Acid Diesters via Organo-Catalyzed Self-Coupling of Propiolates. Tetrahedron Lett..

[32] Zhou L.-H., Yu X.-Q., Pu L. (2010). Reactivity of a PropiolateDimer with Nucleophiles and an Efficient Synthesis of Dimethyl α-Aminoadipate. Tetrahedron Lett..

