# Supplementary material for: α(δ')-Michael Addition of Alkyl Amines to Dimethyl (E)-hex-2-en-4-ynedioate: Synthesis of α,β-Dehydroamino Acid Derivatives
Source: Molecules. 2013 Feb 27;18(3):2611–22. doi: 10.3390/molecules18032611 (PMC6270175; doi:10.3390/molecules18032611)

Supporting Information

Figure S1. ^{1}H-NMR spectrum of compound **(2*E*,4*E*)-4a** (300 MHz, $CDCl_3$).

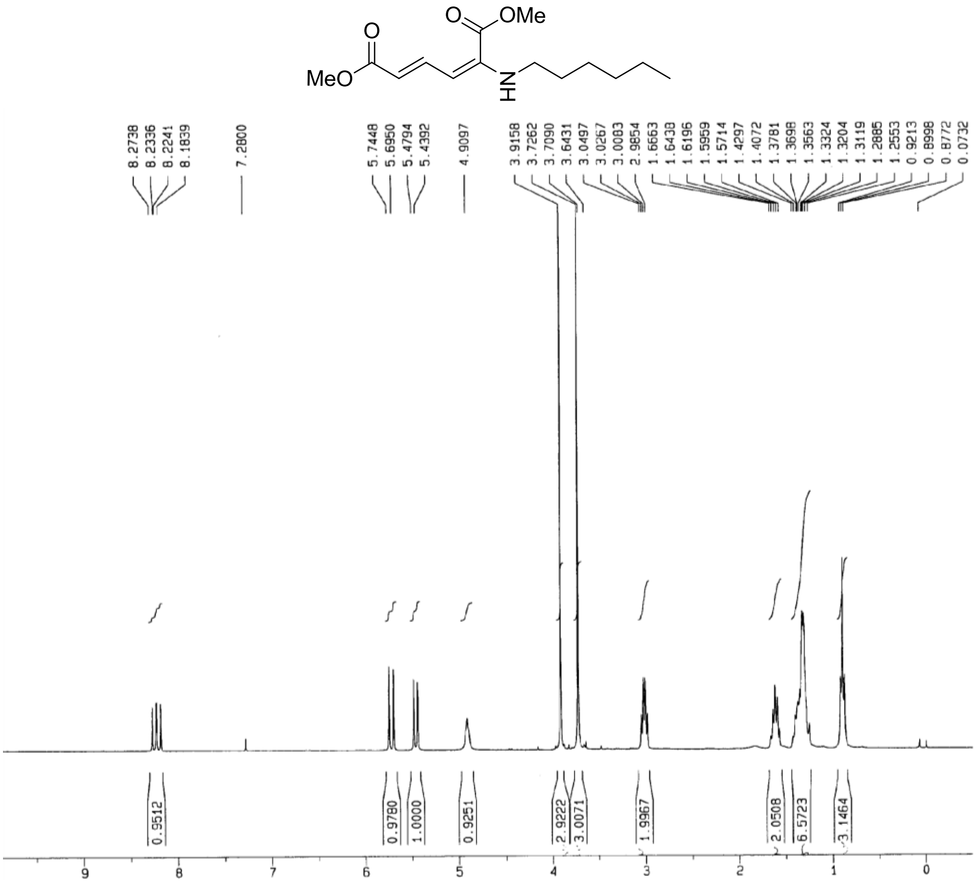

Figure S2. ^{13}C-NMR spectrum of compound **(2*E*,4*E*)-4a** (75 MHz, $CDCl_3$).

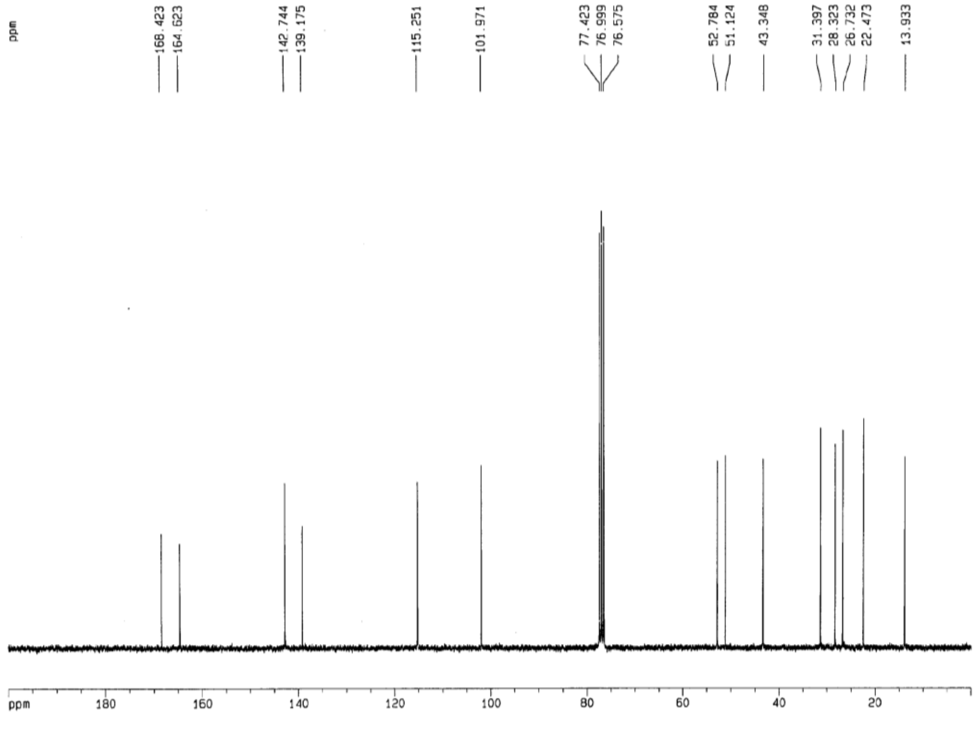

Figure S3. ^{1}H-NMR spectrum of compound **(2*E*,4*E*)-4b** (300 MHz, $CDCl_3$).

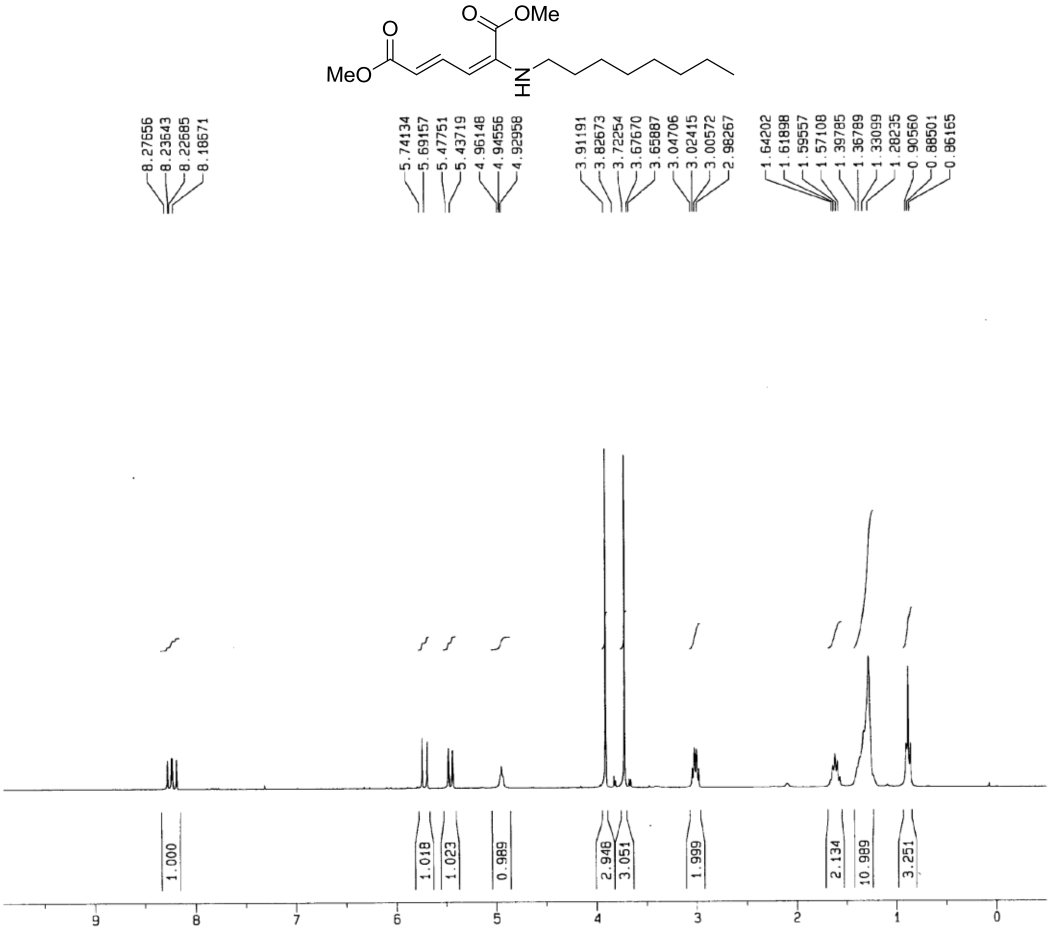

Figure S4. ^{13}C-NMR spectrum of compound **(2*E*,4*E*)-4b** (75 MHz, $CDCl_3$).

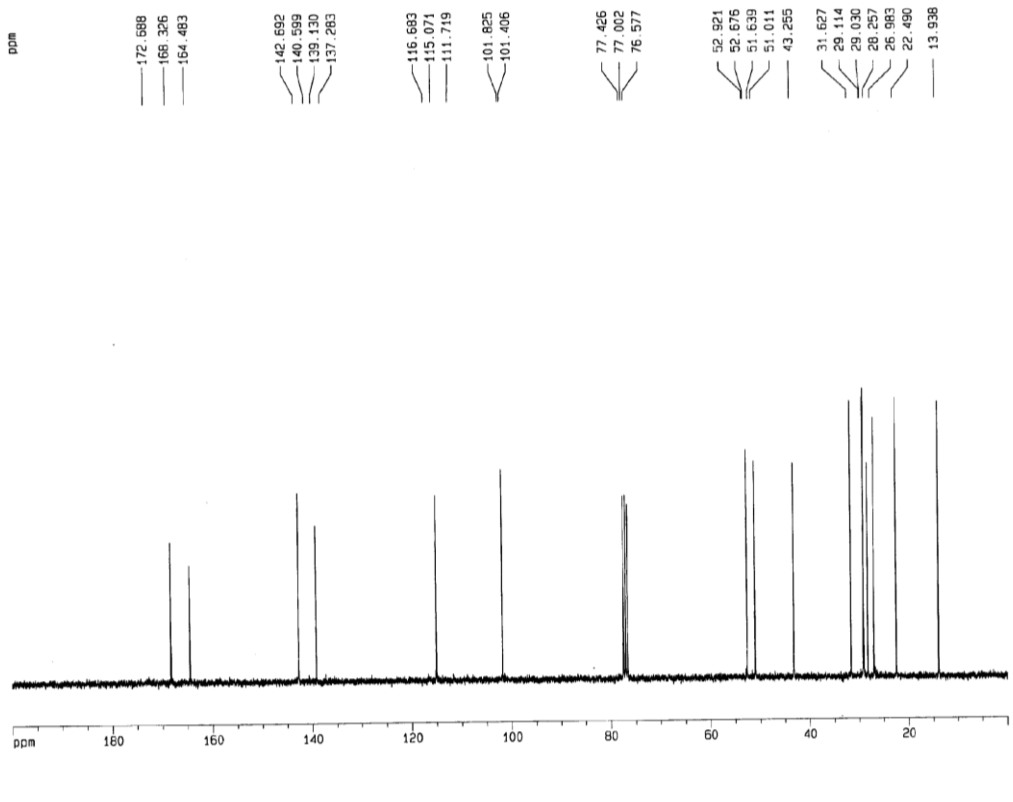

Figure S5. ¹H-NMR spectrum of compound **(2*E*,4*E*)-4c** (300 MHz, $CDCl_3$).

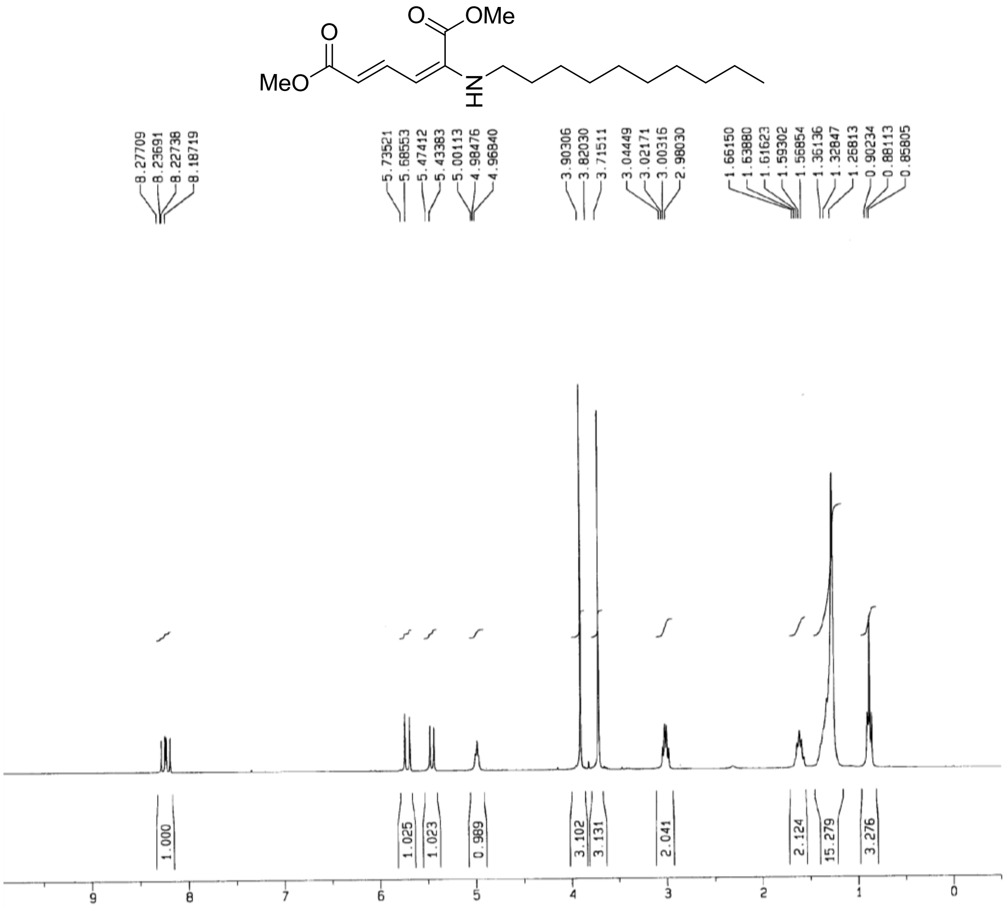

Figure S6. ¹³C-NMR spectrum of compound **(2*E*,4*E*)-4c** (75 MHz, $CDCl_3$).

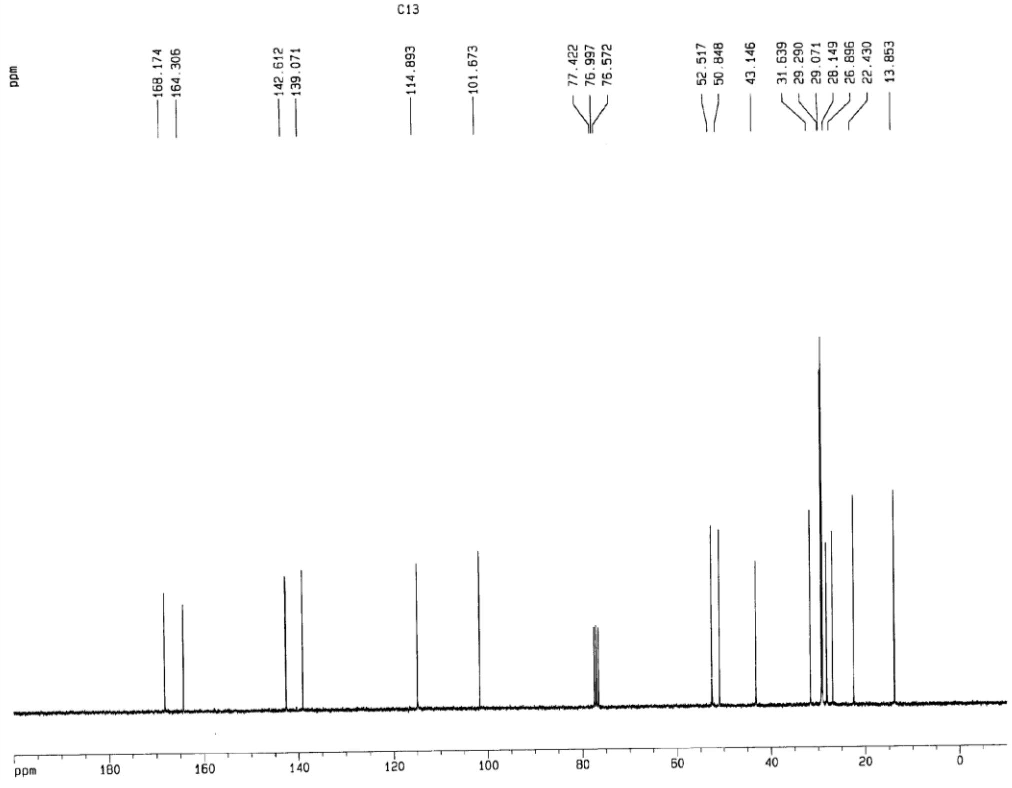

Figure S7. ^{1}H-NMR spectrum of compound **(2*E*,4*E*)-4d** (300 MHz, $CDCl_3$).

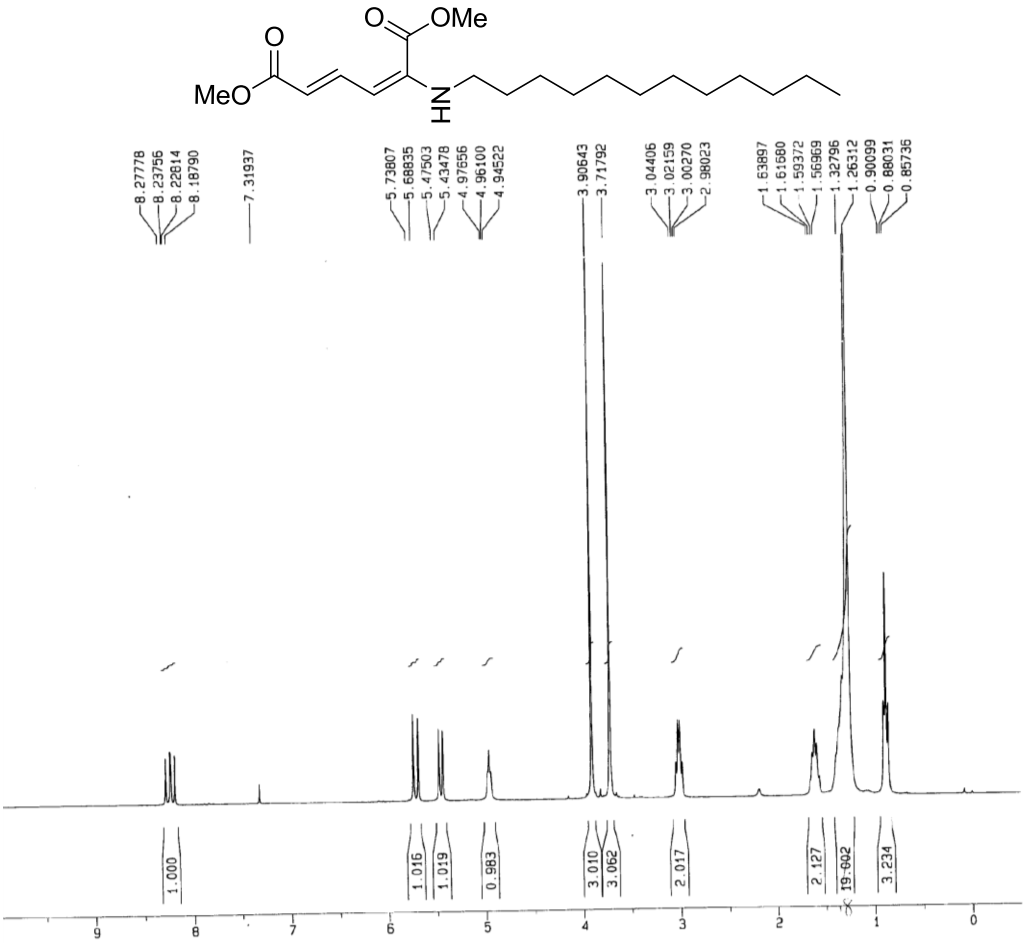

Figure S8. ^{13}C-NMR spectrum of compound **(2*E*,4*E*)-4d** (75 MHz, $CDCl_3$)

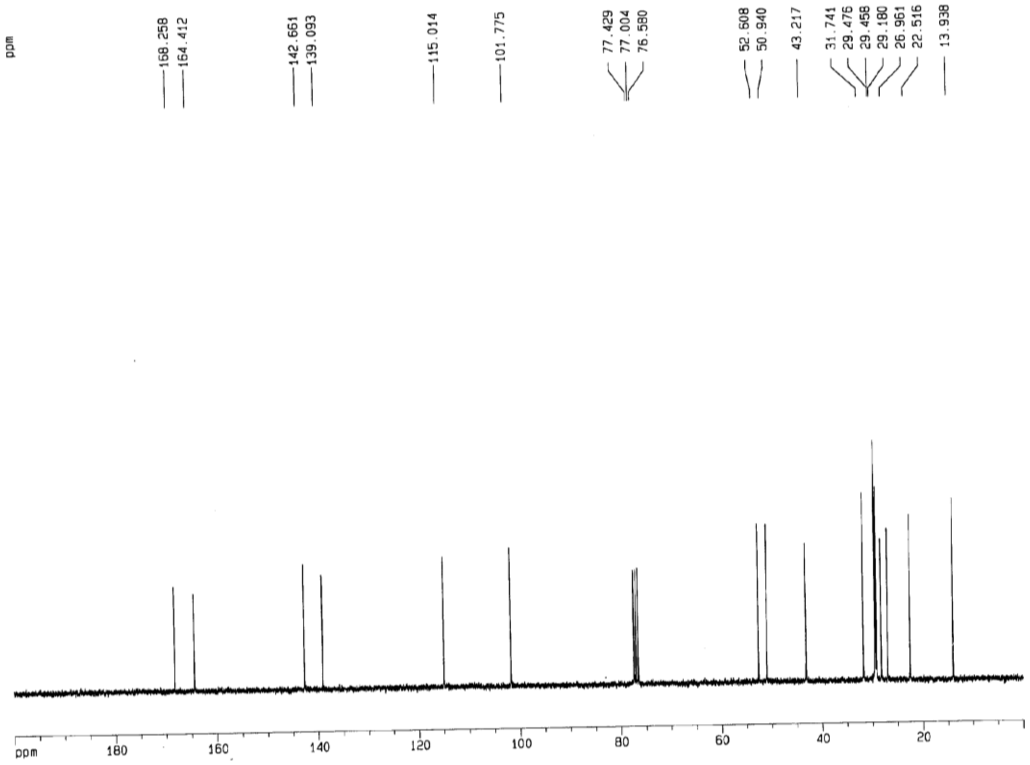

Figure S9. ^{1}H-NMR spectrum of compound **(2*E*,4*E*)-4f** (300 MHz, $CDCl_3$).

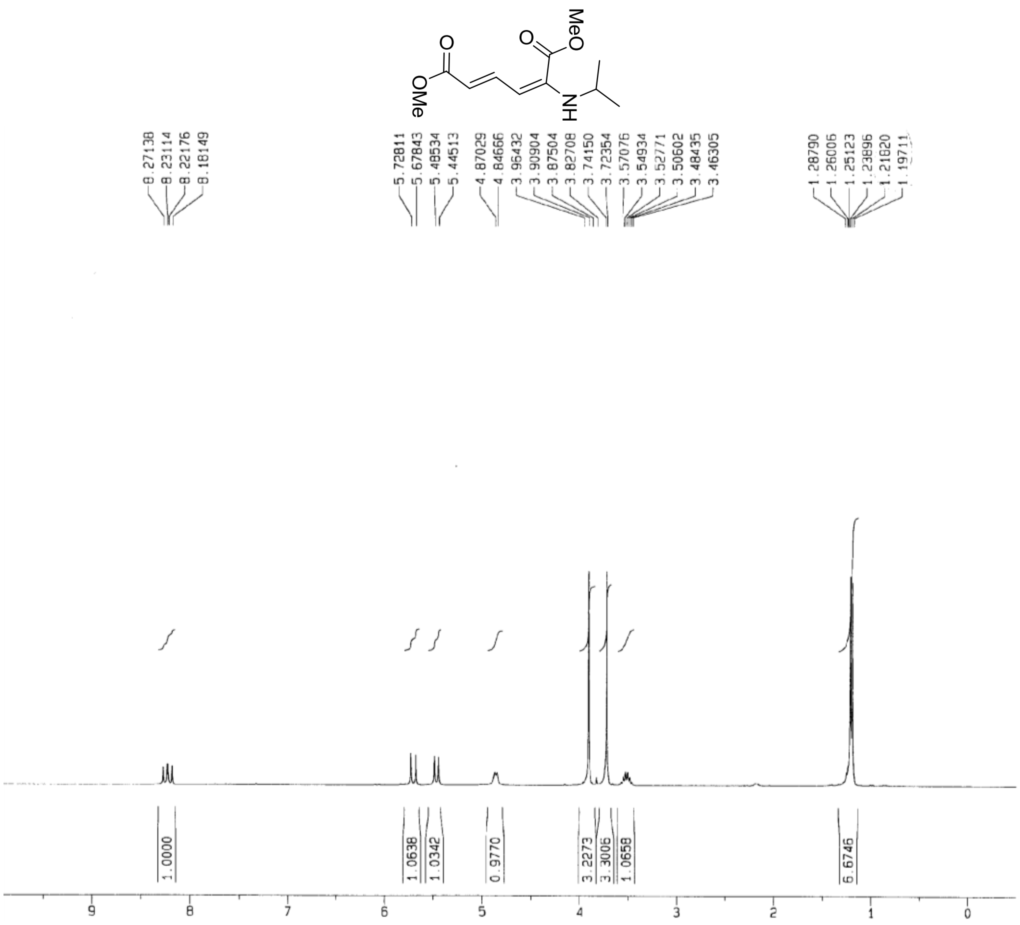

Figure S10. ^{13}C-NMR spectrum of compound **(2*E*,4*E*)-4f** (75 MHz, $CDCl_3$).

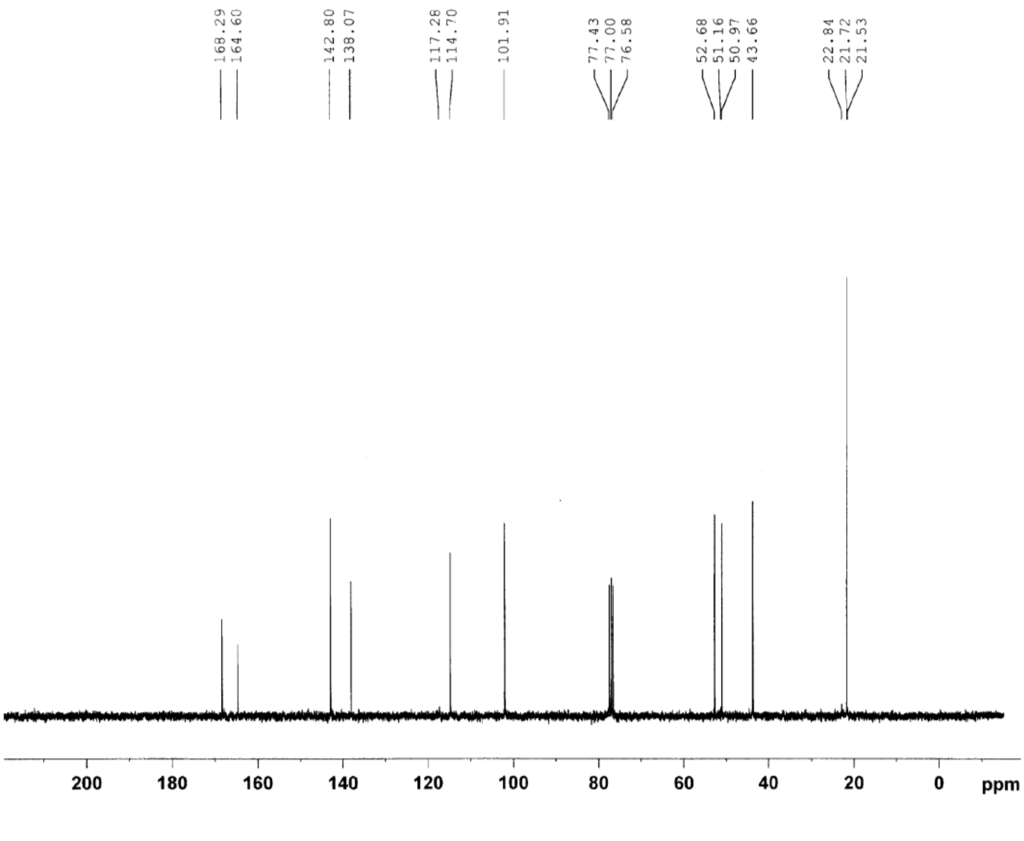

Figure S11. ^{1}H-NMR spectrum of compound **(2*E*,4*E*)-4g** (300 MHz, $CDCl_3$).

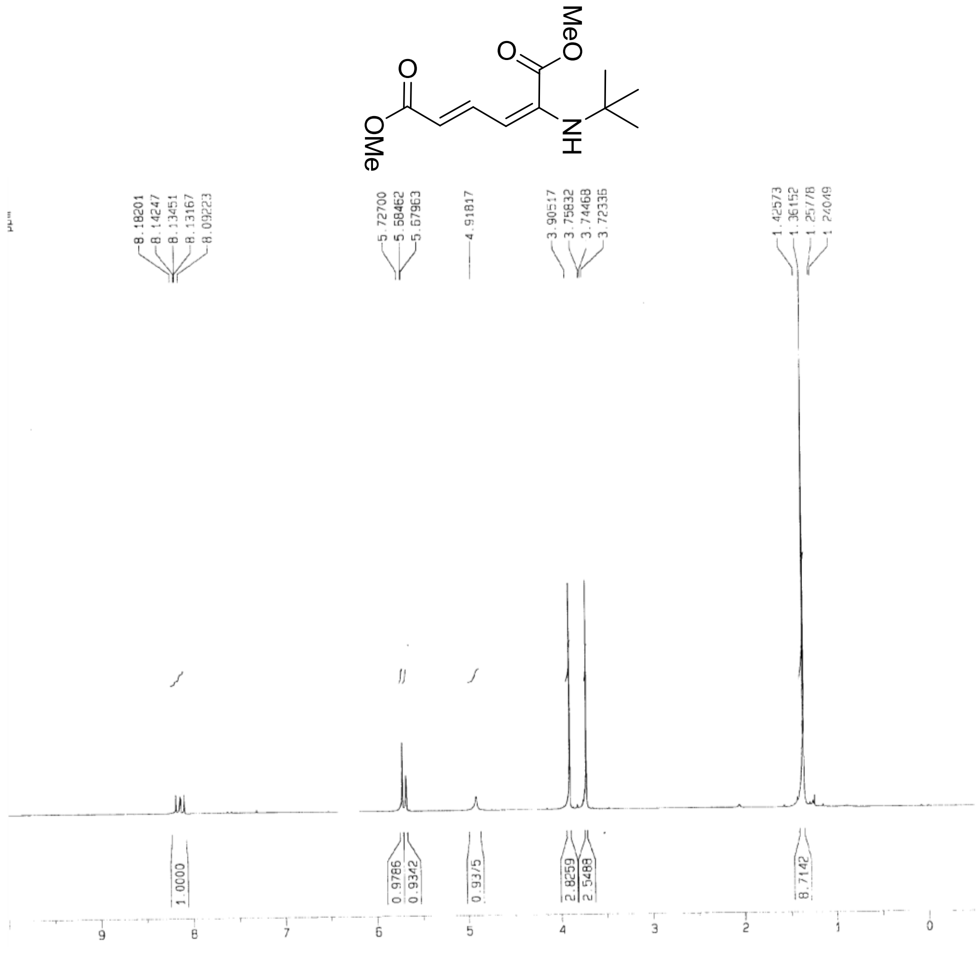

Figure S12. ^{13}C-NMR spectrum of compound **(2*E*,4*E*)-4g** (75 MHz, $CDCl_3$).

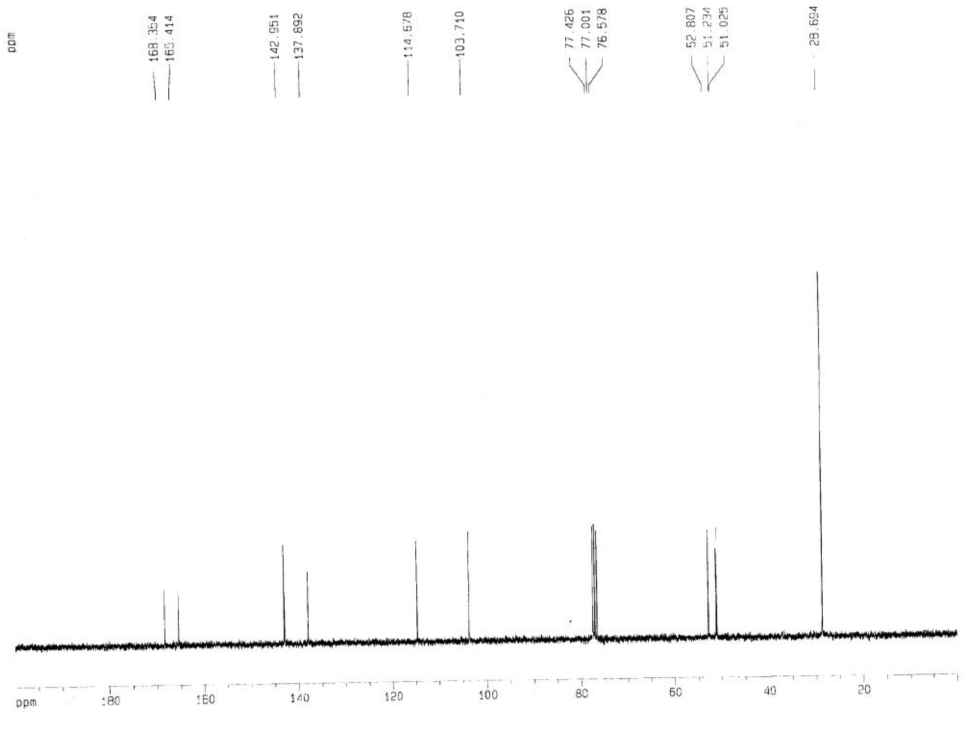

Figure S13. ^{1}H-NMR spectrum of compound **(2*E*,4*E*)-4h** (300 MHz, $CDCl_3$).

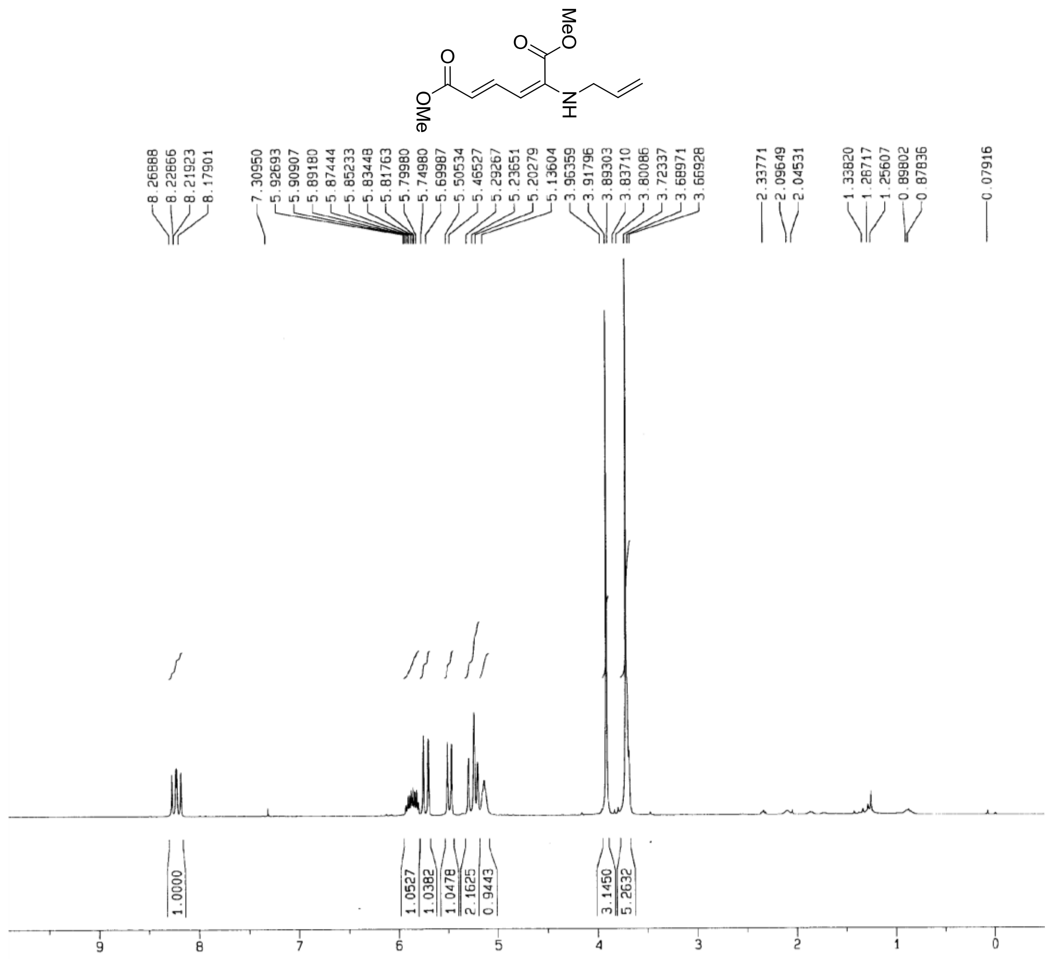

Figure S14. ^{13}C-NMR spectrum of compound **(2*E*,4*E*)-4h** (75 MHz, $CDCl_3$).

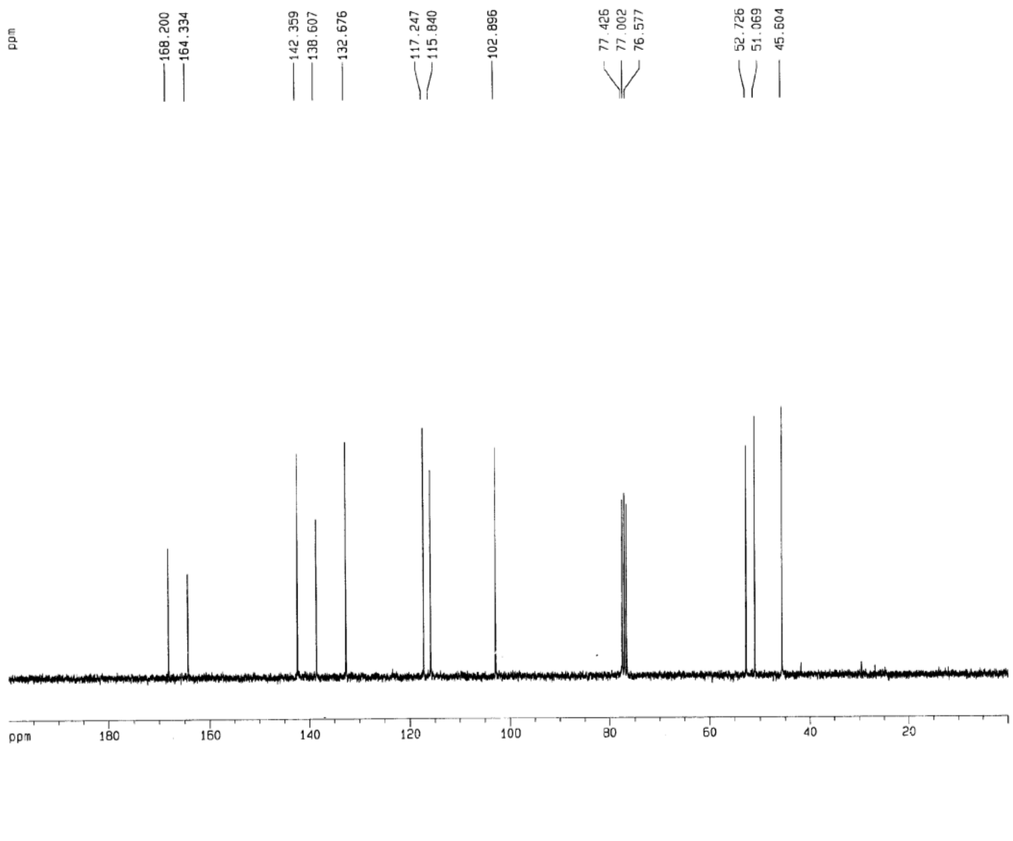

Figure S15. ^{1}H-NMR spectrum of compound **(2*E*,4*E*)-4i** (300 MHz, $CDCl_3$).

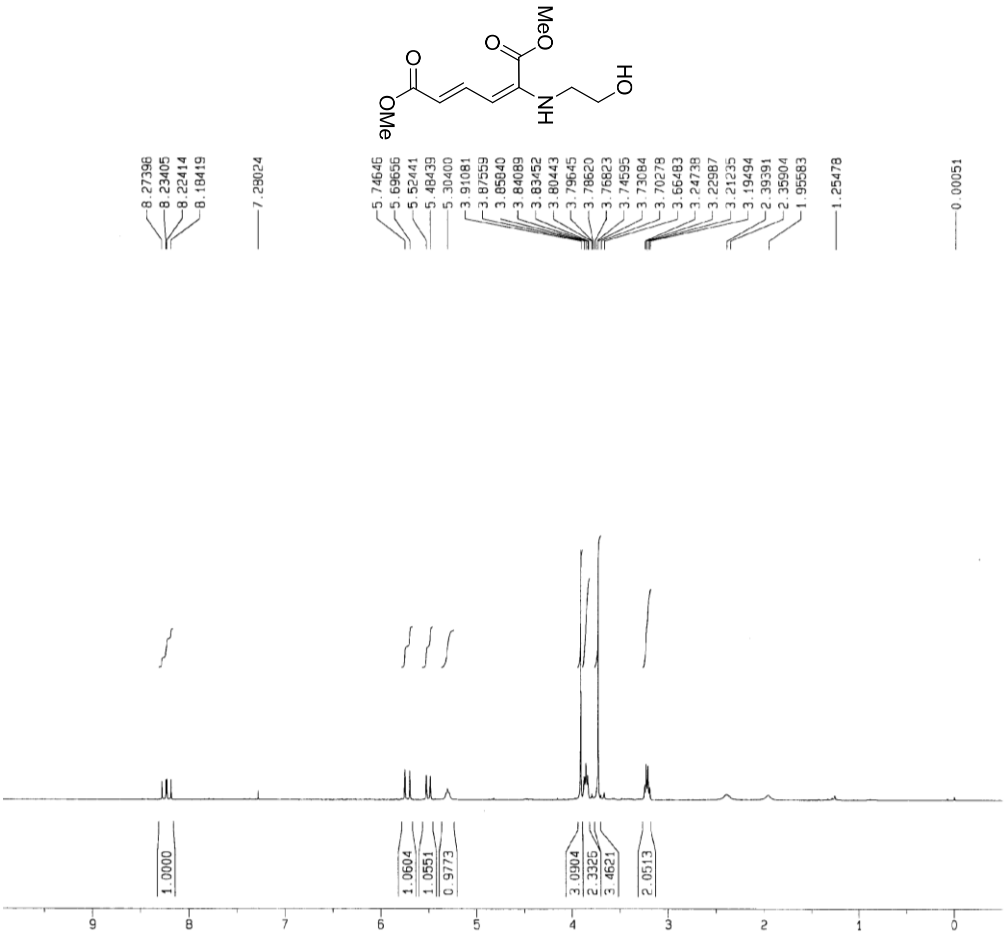

Figure S16. ^{13}C-NMR spectrum of compound **(2*E*,4*E*)-4i** (75 MHz, $CDCl_3$).

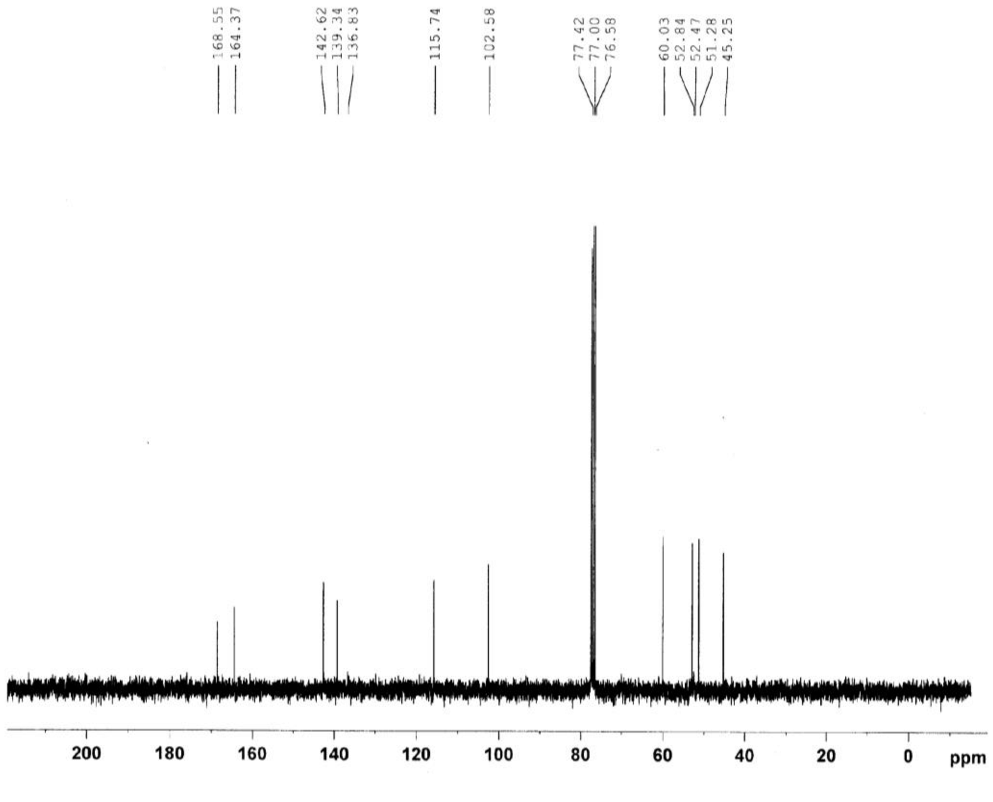

Figure S17. ^{1}H-NMR spectrum of compound **(2*E*,4*E*)-4j** (300 MHz, $CDCl_3$).

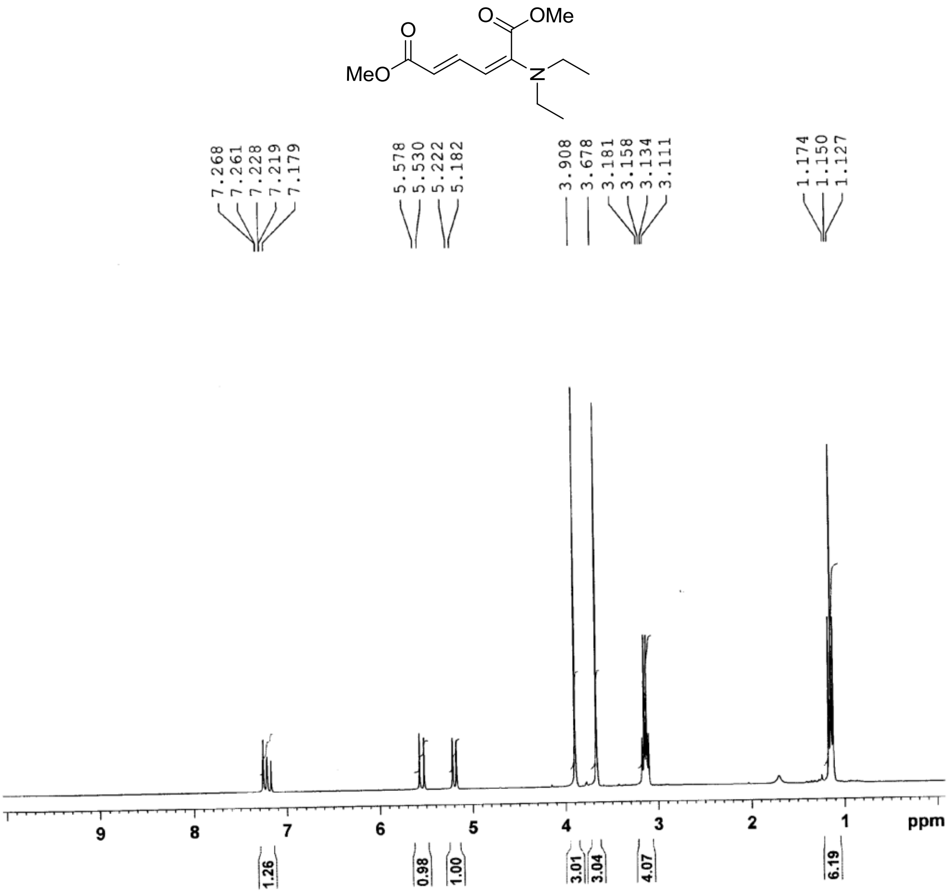

Figure S18. ^{13}C-NMR spectrum of compound **(2*E*,4*E*)-4j** (75 MHz, $CDCl_3$).

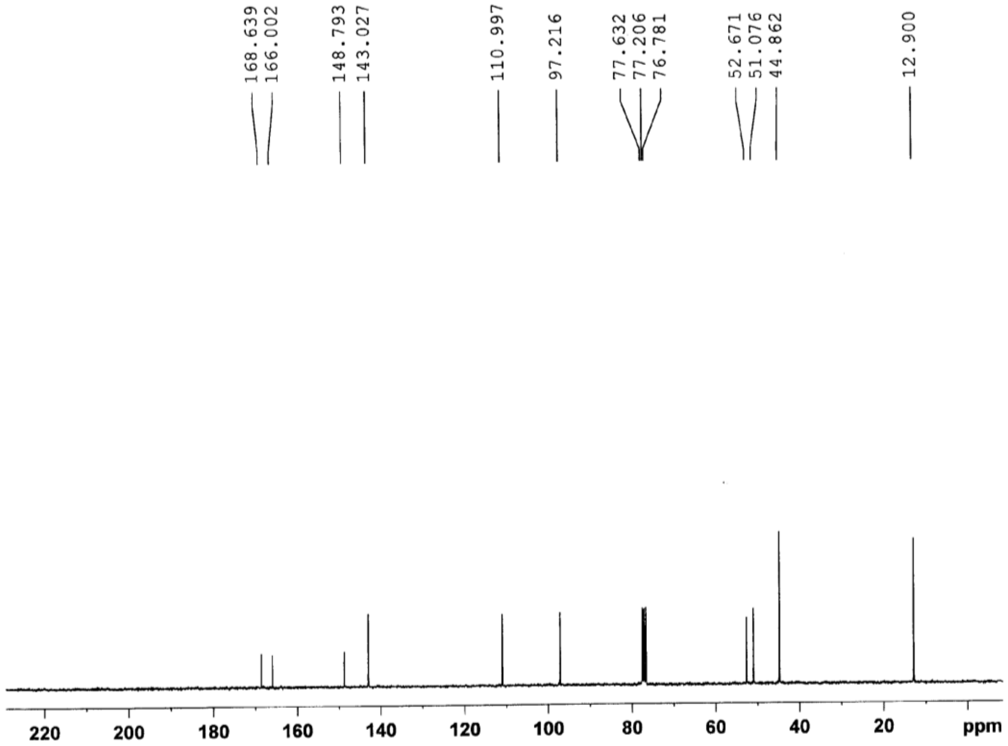

Figure S19. ^{1}H-NMR spectrum of compound **(2*Z*,4*E*)-4j** (300 MHz, $CDCl_3$).

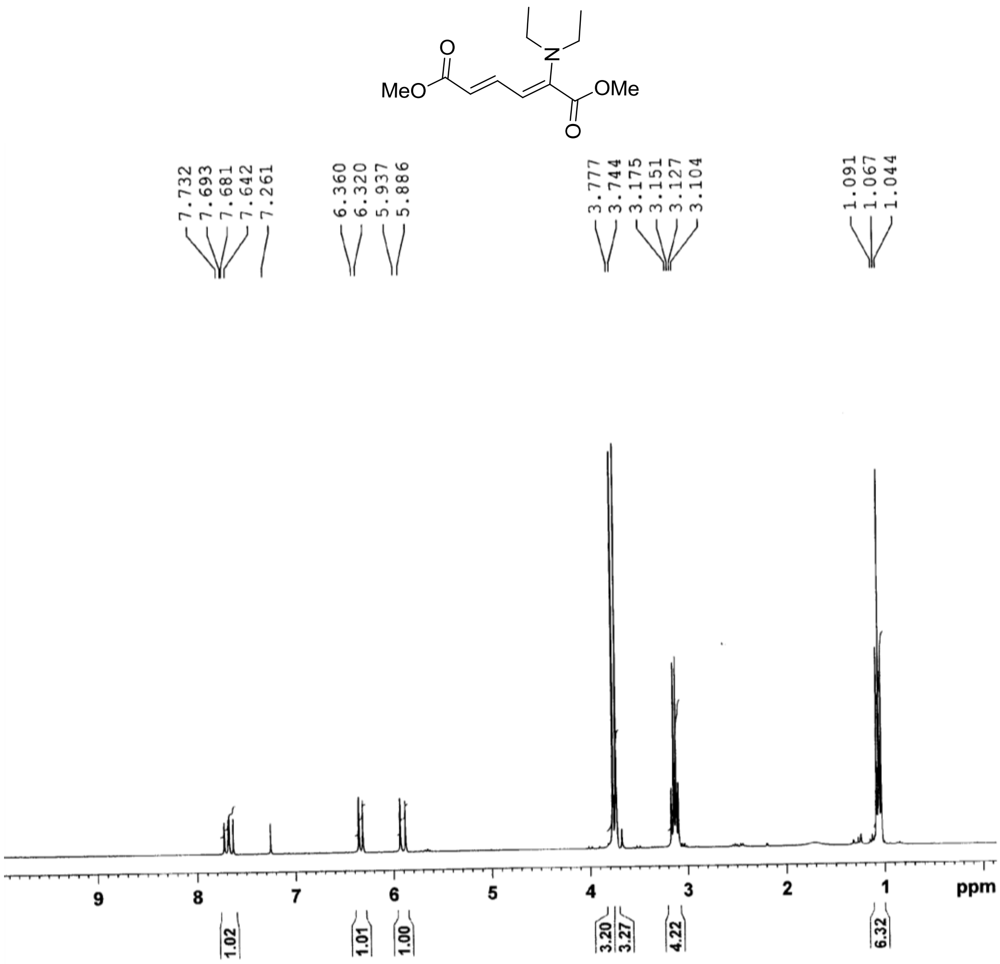

Figure S20. ^{13}C-NMR spectrum of compound **(2*Z*,4*E*)-4j** (75 MHz, $CDCl_3$).

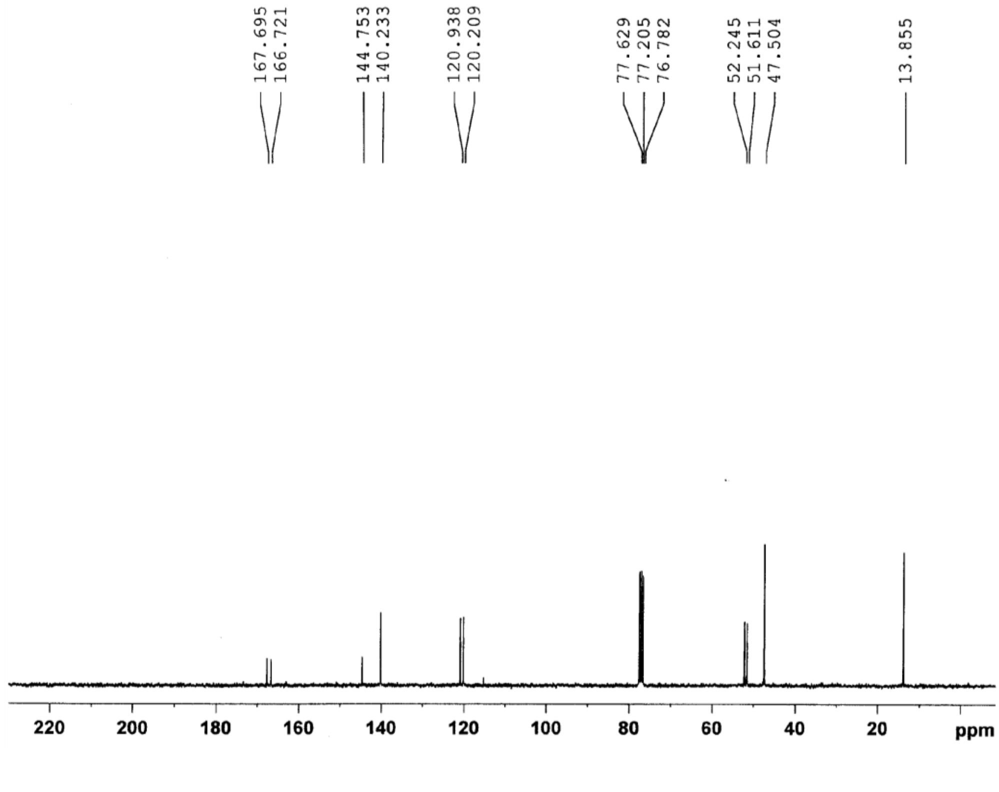

Figure S21. ^{1}H-NMR spectrum of compound **(2*E*,4*E*)-4k** (300 MHz, $CDCl_3$).

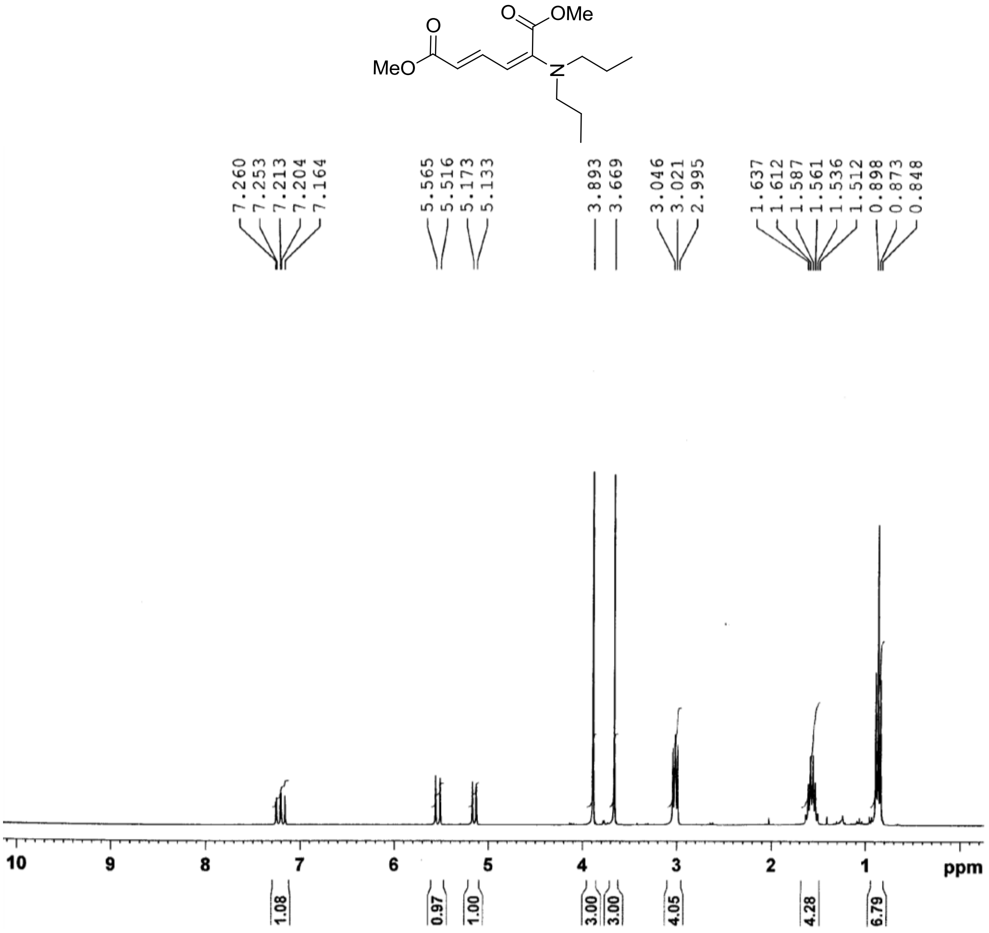

Figure S22. ^{13}C-NMR spectrum of compound **(2*E*,4*E*)-4k** (75 MHz, $CDCl_3$).

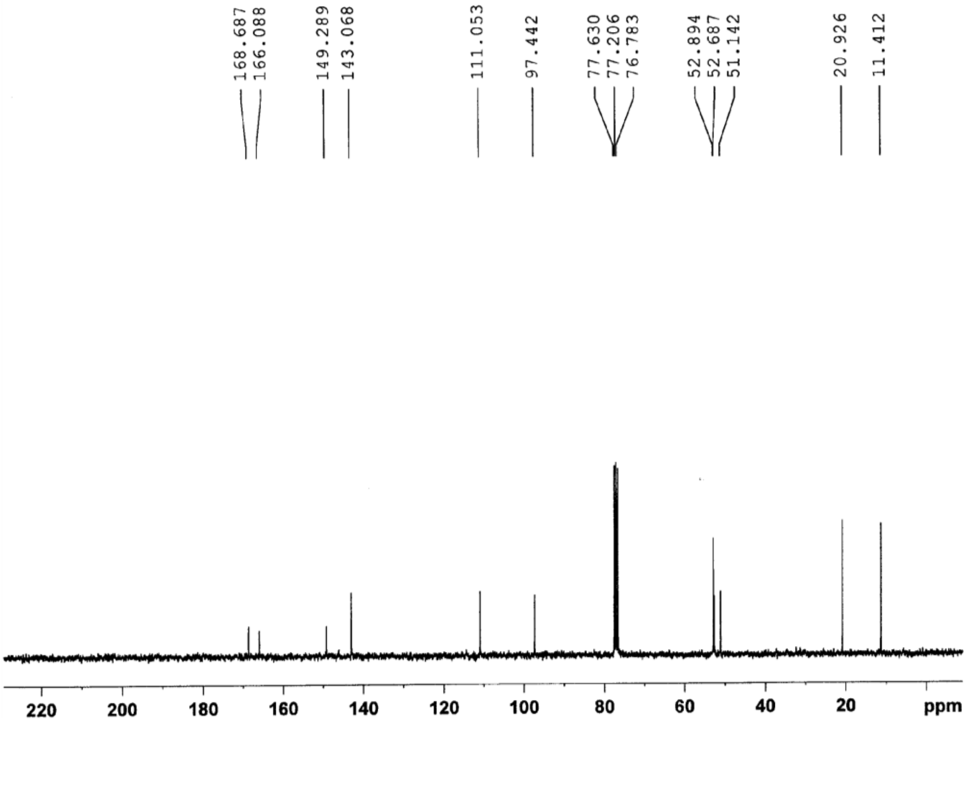

Figure S23. ^{1}H-NMR spectrum of compound **(2*Z*,4*E*)-4k** (300 MHz, $CDCl_3$).

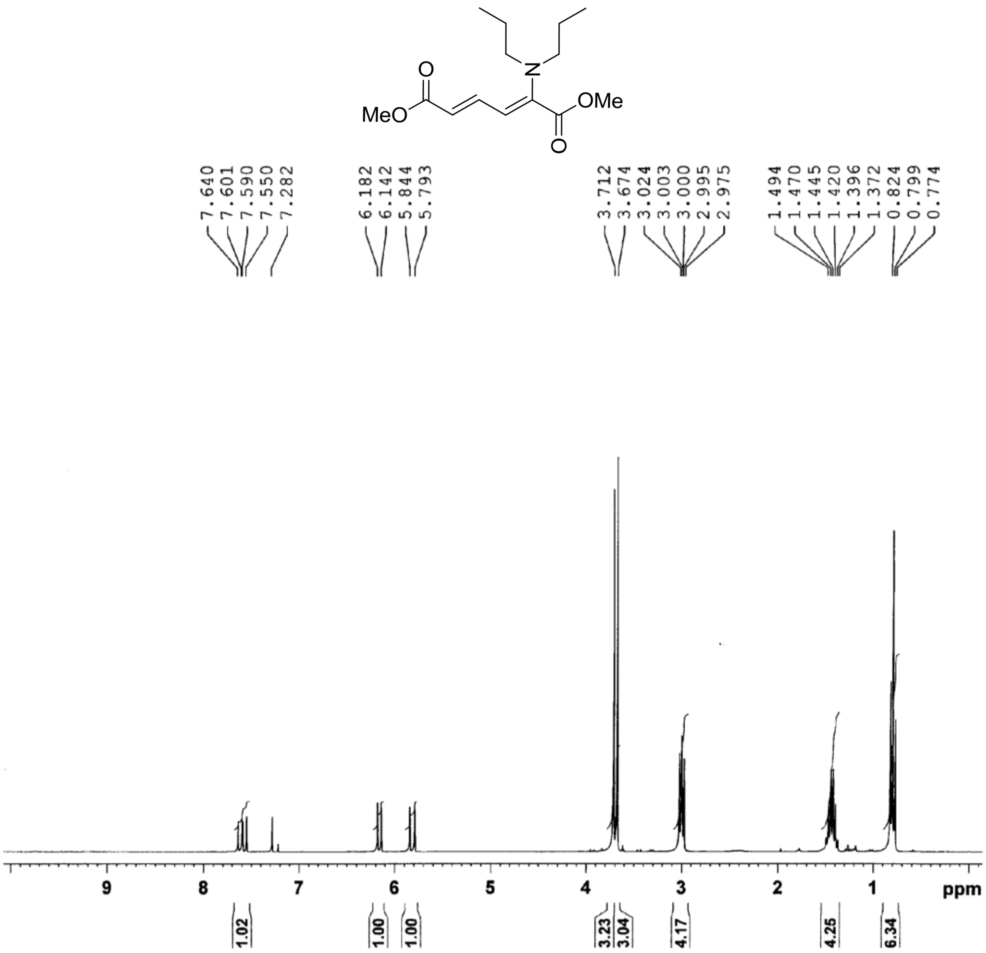

Figure S24. ^{13}C-NMR spectrum of compound **(2*Z*,4*E*)-4k** (75 MHz, $CDCl_3$).

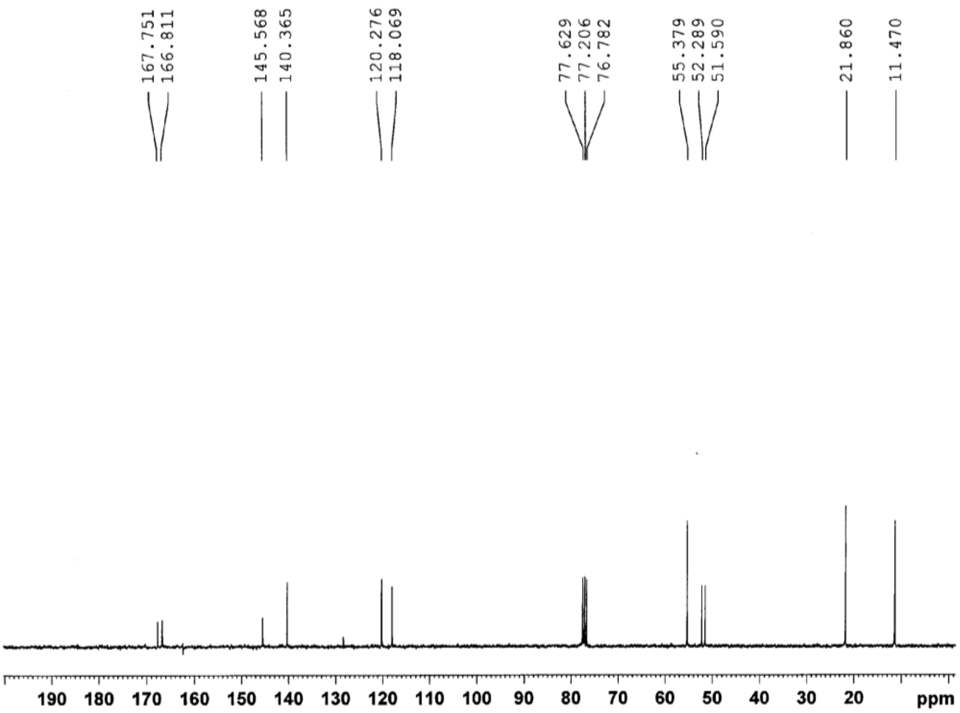

Figure S25. ^{1}H-NMR spectrum of compound **(2*E*,4*E*)-4l** (300 MHz, $CDCl_3$).

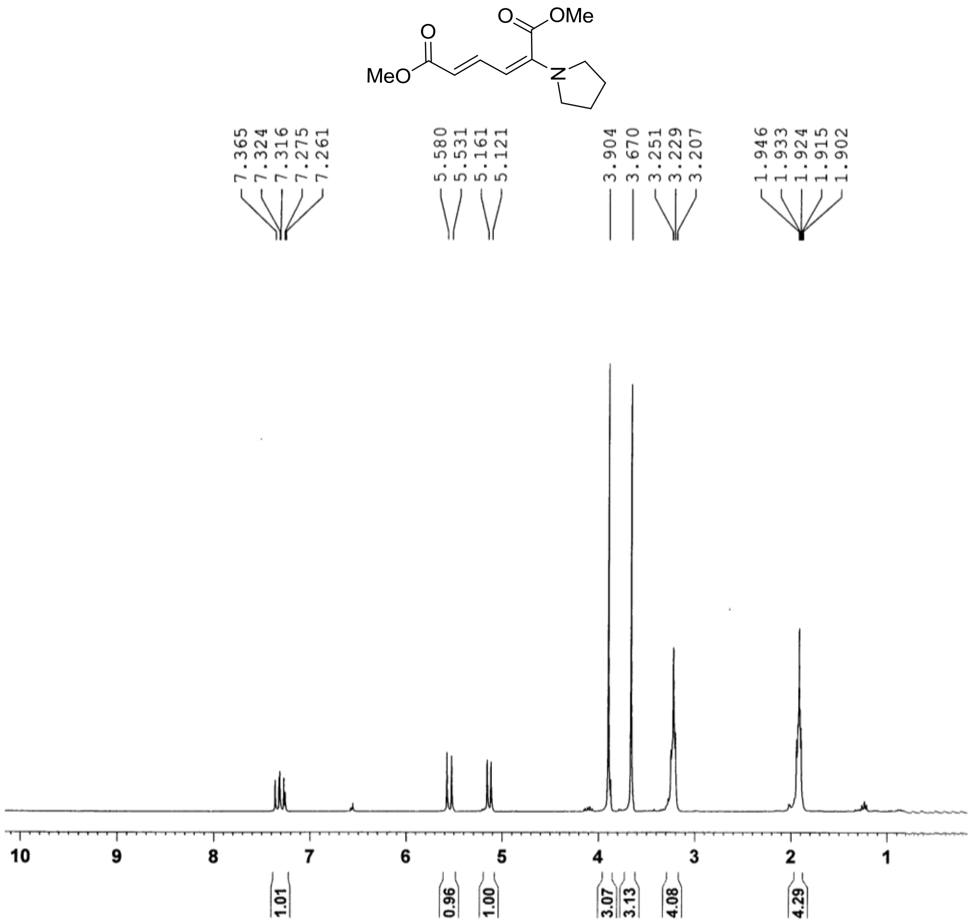

Figure S26. ^{13}C-NMR spectrum of compound **(2*E*,4*E*)-4l** (75 MHz, $CDCl_3$).

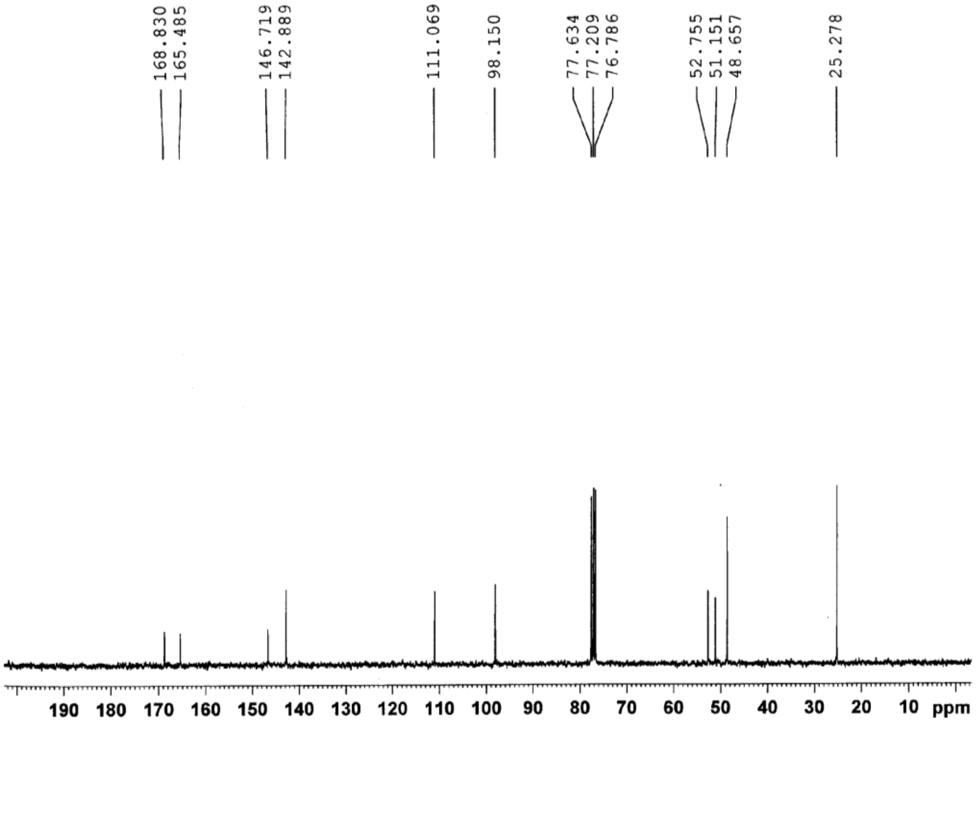

Figure S27. ^{1}H-NMR spectrum of compound **(2*Z*,4*E*)-4l** (300 MHz, $CDCl_3$).

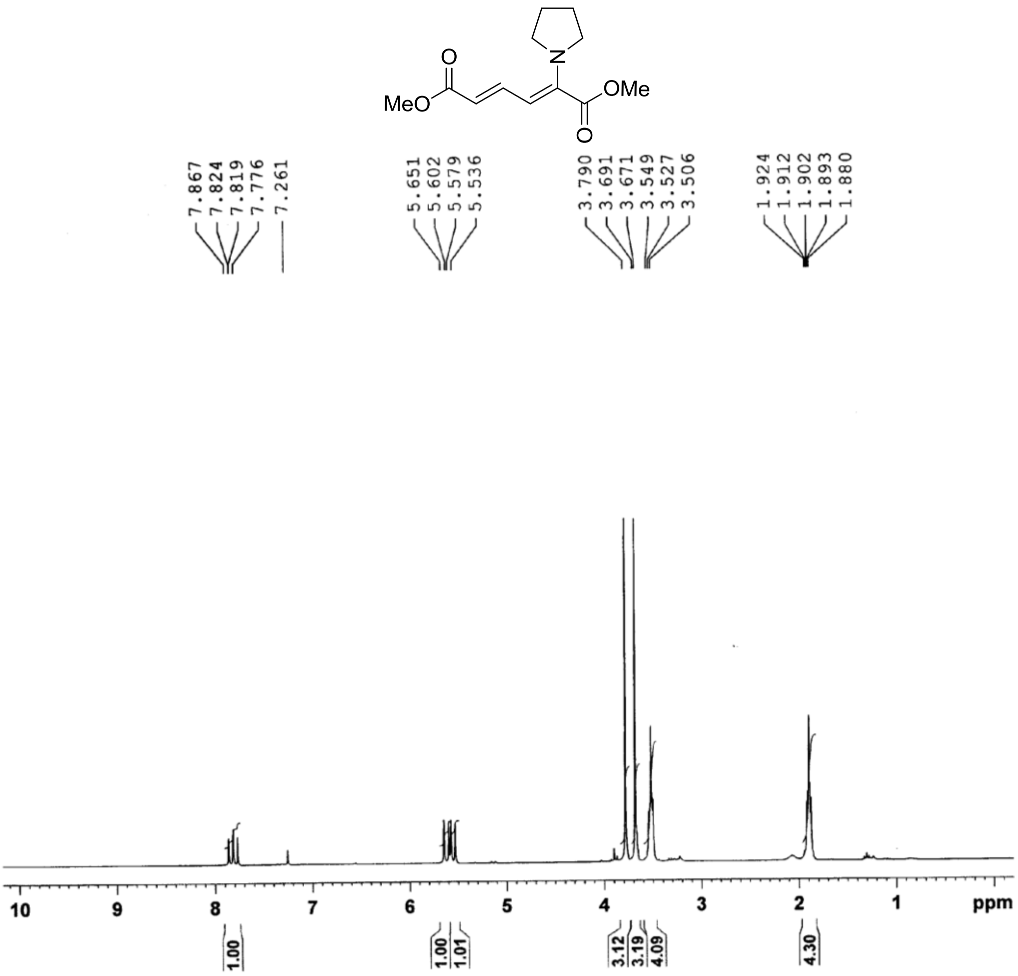

Figure S28. ^{13}C-NMR spectrum of compound **(2*Z*,4*E*)-4l** (75 MHz, $CDCl_3$).

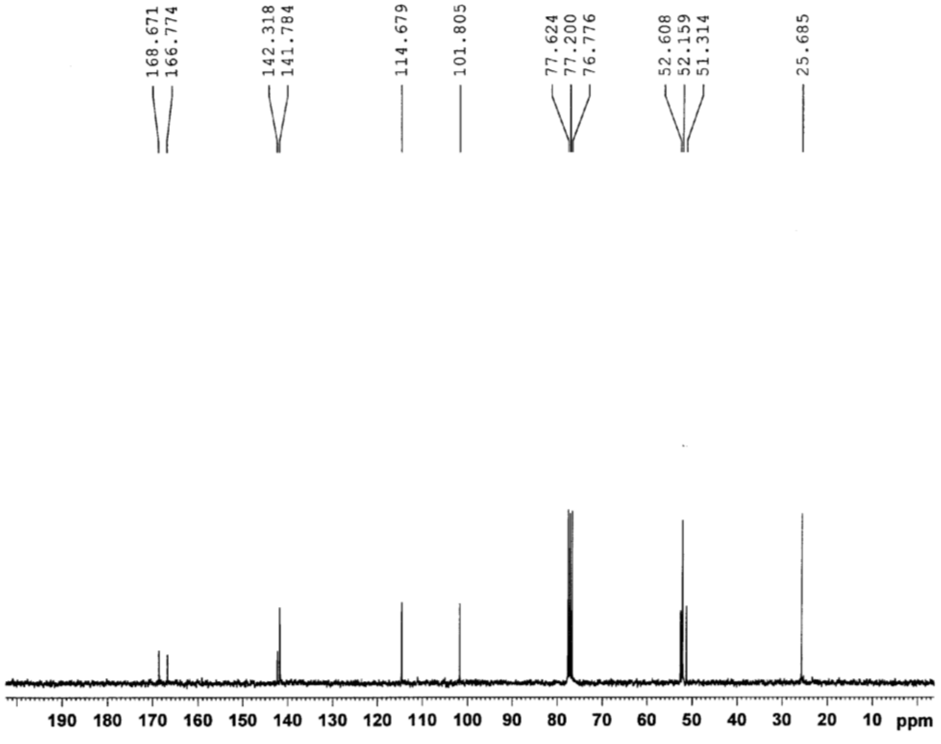

Figure S29. ^{1}H-NMR spectrum of compound **(2*E*,4*E*)-4m** (300 MHz, $CDCl_3$).

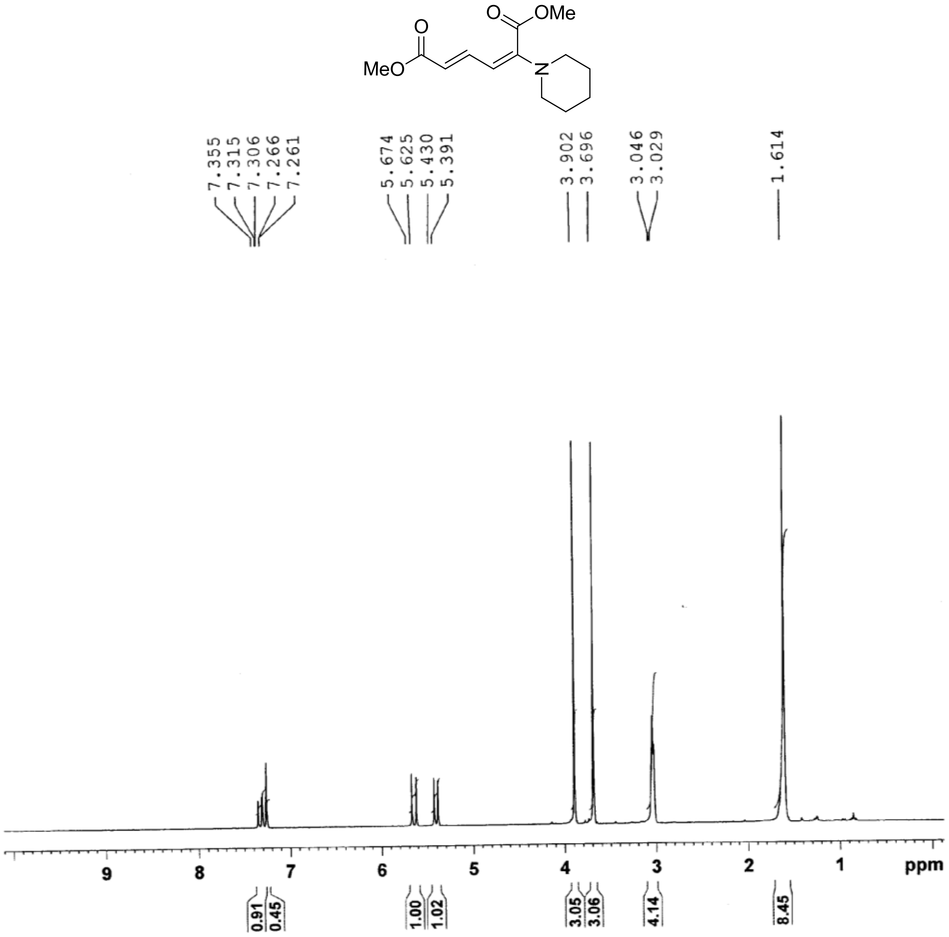

Figure S30. ^{13}C-NMR spectrum of compound **(2*E*,4*E*)-4m** (75 MHz, $CDCl_3$).

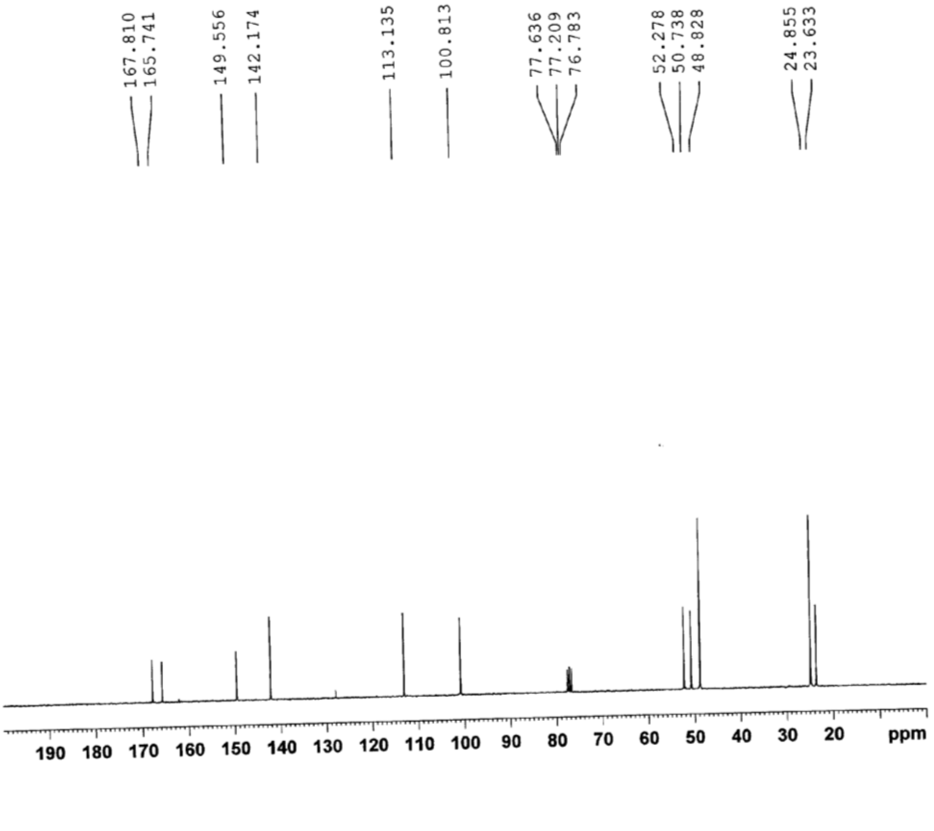

Figure S31. ^{1}H-NMR spectrum of compound **(2*Z*,4*E*)-4m** (300 MHz, $CDCl_3$).

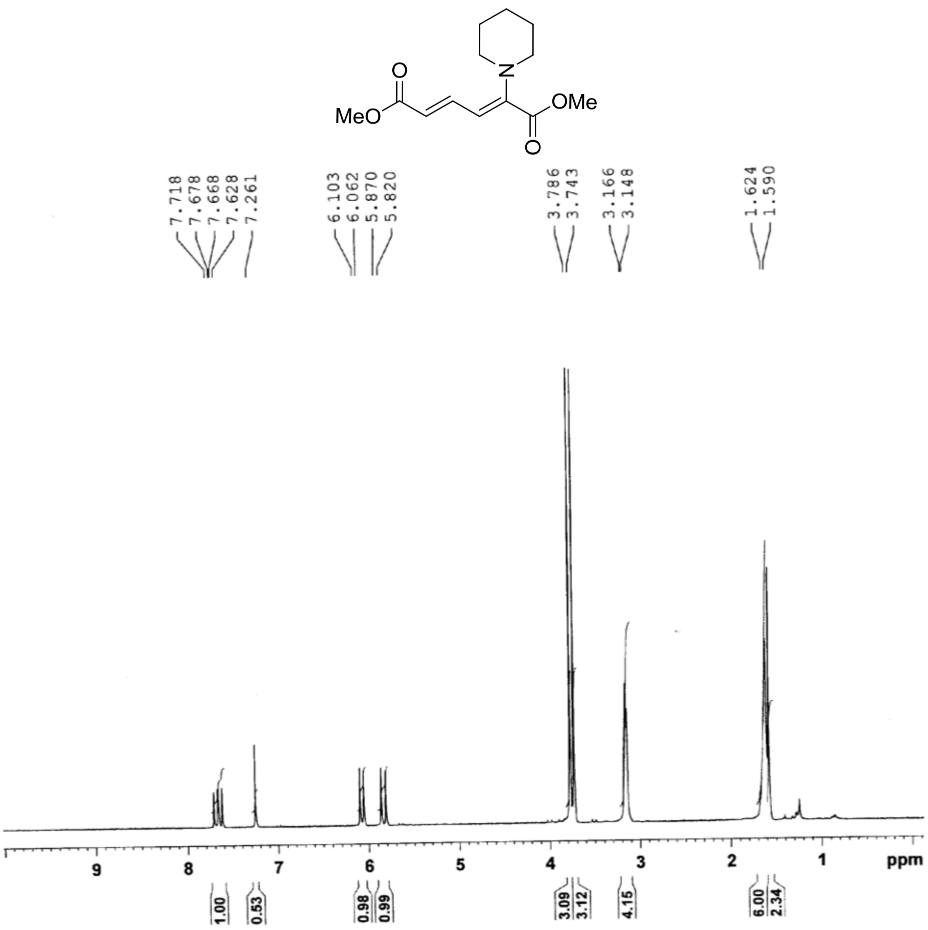

Figure S32. ^{13}C-NMR spectrum of compound **(2*Z*,4*E*)-4m** (75 MHz, $CDCl_3$).

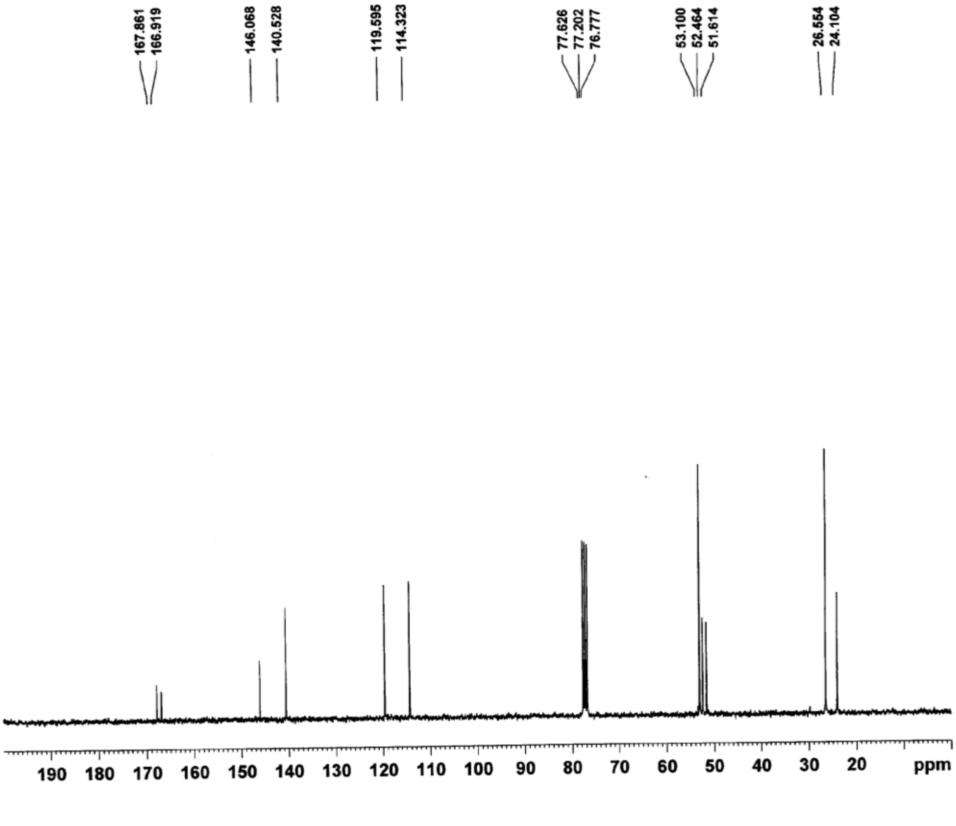

Figure S33. ^{1}H-NMR spectrum of compound **(2*E*,4*E*)-4n** (300 MHz, $CDCl_3$).

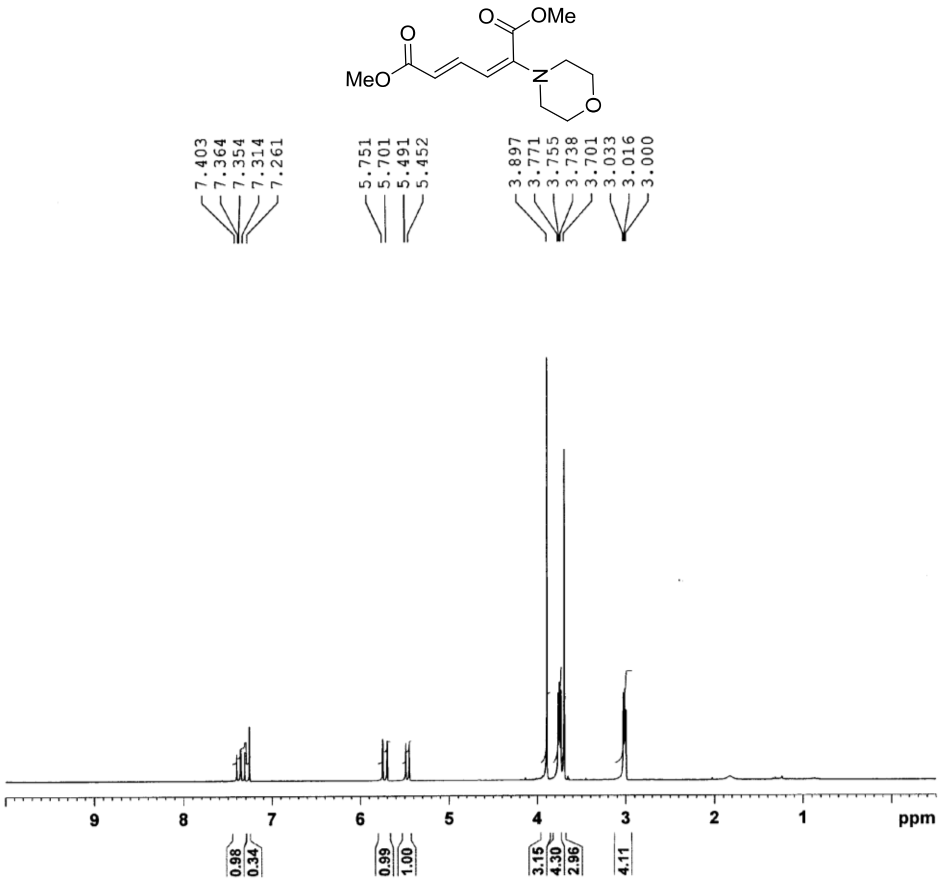

Figure S34. ^{13}C-NMR spectrum of compound **(2*E*,4*E*)-4n** (75 MHz, $CDCl_3$).

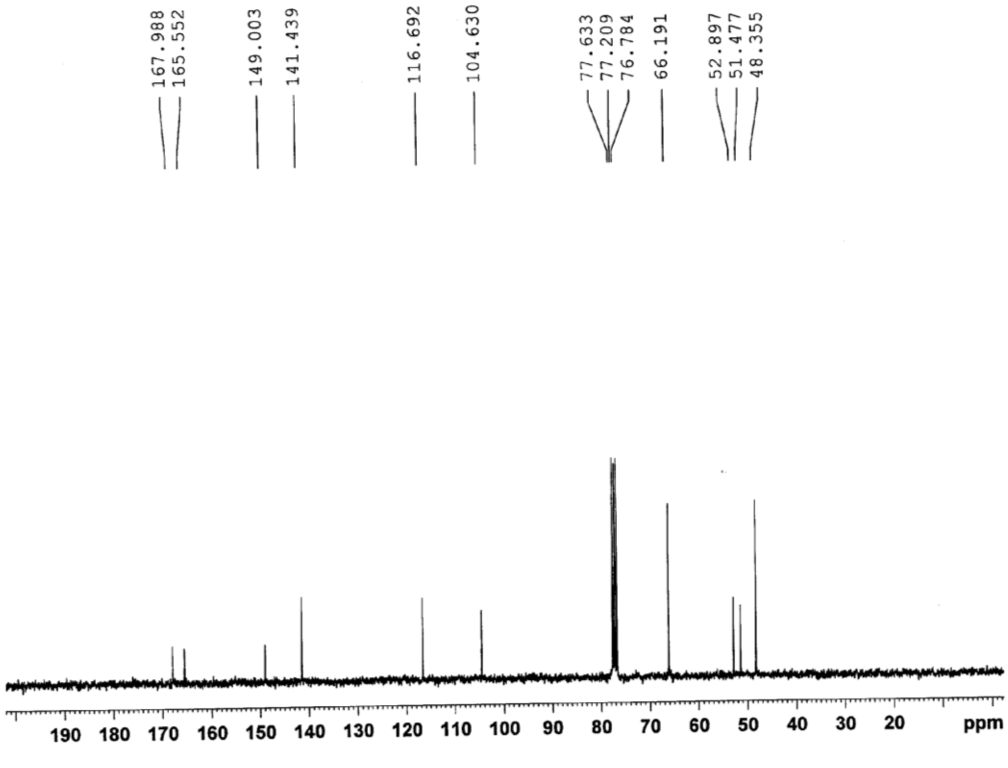

Figure S35. ^{1}H-NMR spectrum of compound **(2*Z*,4*E*)-4n** (300 MHz, $CDCl_3$).

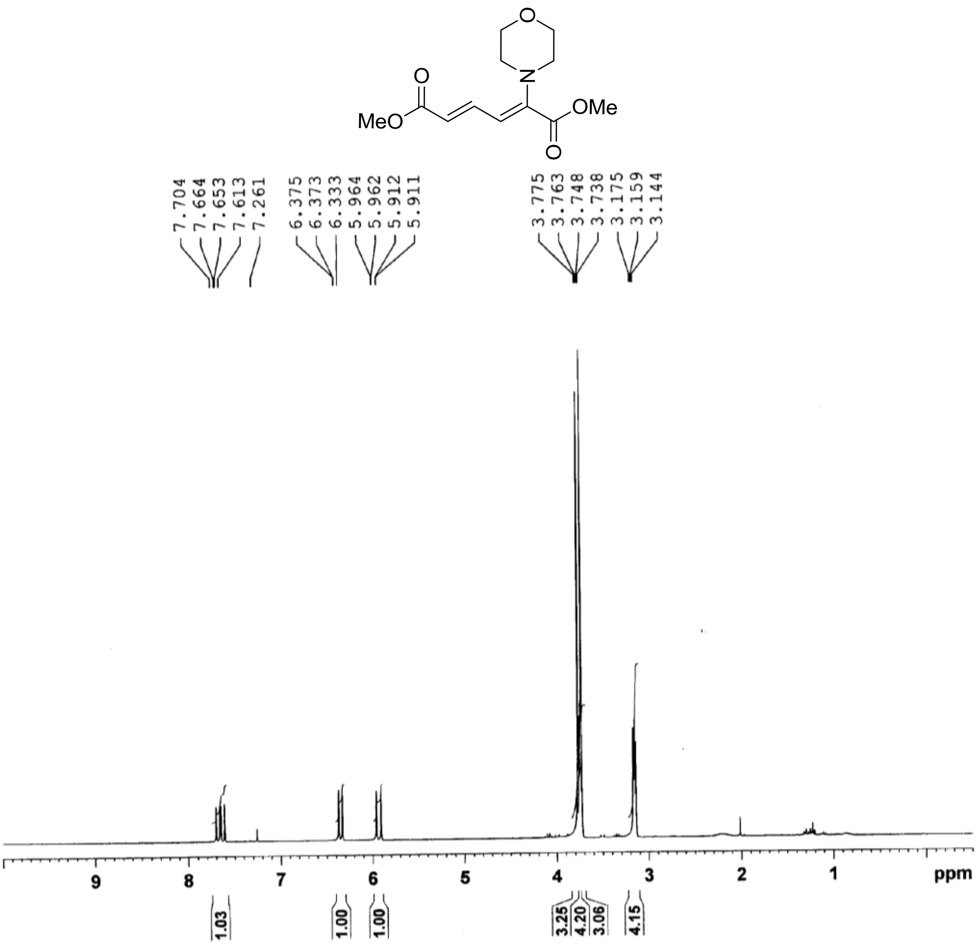

Figure S36. ^{13}C-NMR spectrum of compound **(2*Z*,4*E*)-4n** (75 MHz, $CDCl_3$).

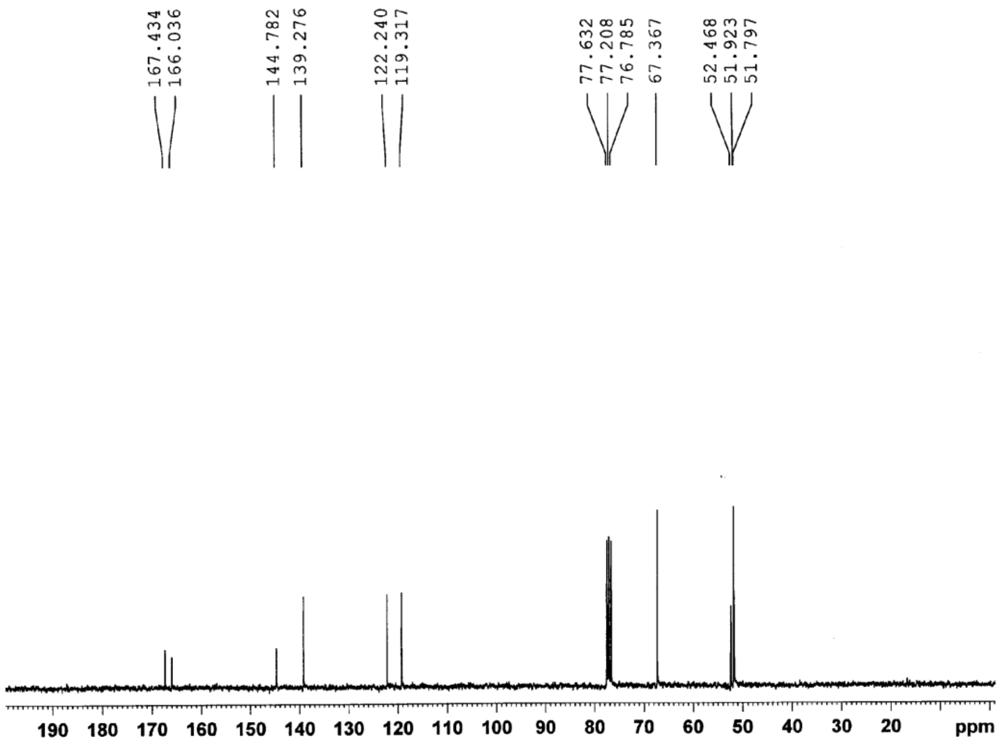

Supplement: Supplementary file 1 [file molecules-18-02611-s001.pdf]
